# CRISPR/Cas9 screen in human iPSC-derived cortical neurons identifies NEK6 as a novel disease modifier of *C9orf72* poly(PR) toxicity

**DOI:** 10.1002/alz.12760

**Published:** 2022-08-22

**Authors:** Wenting Guo, Haibo Wang, Arun Kumar Tharkeshwar, Julien Couthouis, Elke Braems, Pegah Masrori, Evelien Van Schoor, Yannan Fan, Karan Ahuja, Matthieu Moisse, Maarten Jacquemyn, Rodrigo Furtado Madeiro da Costa, Madhavsai Gajjar, Sriram Balusu, Tine Tricot, Laura Fumagalli, Nicole Hersmus, Rekin’s Janky, Francis Impens, Pieter Vanden Berghe, Ritchie Ho, Dietmar Rudolf Thal, Rik Vandenberghe, Muralidhar L. Hegde, Siddharthan Chandran, Bart De Strooper, Dirk Daelemans, Philip Van Damme, Ludo Van Den Bosch, Catherine Verfaillie

**Affiliations:** 1 Stem Cell Institute, Department of Devolpment and Regeneration, KU Leuven, Leuven, Belgium; 2 Department of Neurosciences, Experimental Neurology, Laboratory of Neurobiology, KU Leuven-University of Leuven, Leuven, Belgium; 3 VIB, Center for Brain & Disease Research, Leuven, Belgium and Leuven Brain Institute (LBI), Leuven, Belgium; 4 Division of DNA Repair Research, Department of Neurosurgery, Center for Neuroregeneration, Houston Methodist Research Institute, Houston, Texas, USA; 5 Department of Neuroscience Research at Neurological Surgery, Weill Medical College, New York, New York, USA; 6 Department of Genetics, Stanford University School of Medicine, Stanford, California, USA; 7 Department of Neurology, University Hospitals Leuven, Leuven, Belgium; 8 Laboratory of Neuropathology, Department of Imaging and Pathology, KU Leuven, and Leuven Brain Institute (LBI), Leuven, Belgium; 9 KU Leuven Department of Microbiology, Immunology and Transplantation, Laboratory of Virology and Chemotherapy, Rega Institute for Medical Research, Leuven, Belgium; 10 VIB Nucleomics Core, Leuven, Belgium; 11 VIB-UGent Center for Medical Biotechnology, Ghent, Belgium; 12 Department of Biomolecular Medicine, Ghent University, Ghent, Belgium; 13 VIB Proteomics Core, Ghent, Belgium; 14 Translational Research Centre for Gastrointestinal Disorders (TARGID), KU Leuven–University of Leuven, Leuven, Belgium; 15 Center for Neural Science and Medicine, Board of Governors Regenerative Medicine Institute, Departments of Biomedical Sciences and Neurology, Cedars-Sinai Medical Center, Los Angeles, California, USA; 16 KU Leuven-Laboratory for Cognitive Neurology, Department of Neurosciences, Leuven Brain Institute, Leuven, Belgium; 17 UK-Dementia Research Institute at University College London, London, UK; 18 Centre for Clinical Brain Sciences, University of Edinburgh, Edinburgh, UK

**Keywords:** amyotrophic lateral sclerosis, C9orf72, CRISPR/Cas9 screen, DNA damage, frontotemporal dementia, human pluripotent stem cells, NEK6, neurodegeneration, p53, PR toxicity

## Abstract

**Introduction:**

The most common genetic cause of frontotemporal dementia (FTD) and amyotrophic lateral sclerosis (ALS) are hexanucleotide repeats in chromosome 9 open reading frame 72 (*C9orf72*). These repeats produce dipeptide repeat proteins with poly(PR) being the most toxic one.

**Methods:**

We performed a kinome-wide CRISPR/Cas9 knock-out screen in human induced pluripotent stem cell (iPSC) -derived cortical neurons to identify modifiers of poly(PR) toxicity, and validated the role of candidate modifiers using in vitro, in vivo, and ex-vivo studies.

**Results:**

Knock-down of NIMA-related kinase 6 (NEK6) prevented neuronal toxicity caused by poly(PR). Knock-down of nek6 also ameliorated the poly(PR)-induced axonopathy in zebrafish and NEK6 was aberrantly expressed in *C9orf72* patients. Suppression of NEK6 expression and NEK6 activity inhibition rescued axonal transport defects in cortical neurons from *C9orf72* patient iPSCs, at least partially by reversing p53-related DNA damage.

**Discussion:**

We identified NEK6, which regulates poly(PR)-mediated p53-related DNA damage, as a novel therapeutic target for *C9orf72* FTD/ALS.

## 1 | INTRODUCTION

Frontotemporal dementia (FTD) and amyotrophic lateral sclerosis (ALS) are two devastating neurodegenerative diseases, with clinical, pathological, and genetic overlap^1^ and without an effective treatment.^2^ FTD is the second most common form of dementia after Alzheimer’s disease in patients less than 65 years old.^1^ Neuronal loss in the frontal and temporal cortices results in personality and behavioral changes or gradual impairment of language skills.^1^ ALS is the most common motor neuron degenerative disease in adulthood.^2^ It is characterized by the selective loss of motor neurons in the motor cortex (layer V of the cerebral cortex), brainstem and spinal cord.^2^ This results in cramping of muscles, progressive muscle weakness and atrophy, dysarthria and dysphagia, and death, usually 2–5 years after the onset of symptoms, mostly due to respiratory failure.^2^ Hexanucleotide repeat expansions in *chromosome 9 open reading frame 72* (*C9orf72*) are the most frequent genetic cause of both FTD and ALS.^1^ These expansions consist of a hexanucleotide repeat (GGGGCC or G_4_C_2_) in the 5′ noncoding sequence of this gene.^3^ A gain of toxic function is thought to be involved in FTD/ALS as a result of the protein products derived from the expanded G_4_C_2_ repeat.^3^ These G_4_C_2_ repeats produce sense and antisense RNA, which are translated into dipeptide repeat proteins (DPRs) through an unconventional repeat associated form of non-AUG (RAN) translation.^3^ DPRs translated from six reading frames of both sense (poly-GA, poly-GR, and poly-GP) and antisense (poly-PA, poly-PR, and poly-GP) repeat RNAs are found in *postmortem* central nervous system (CNS) tissue and induced pluripotent stem cell (iPSC) models from FTD/ALS patients carrying a *C9orf72* repeat expansion.^3^ Studies in *Drosophila* and human cells suggested that DPRs are the main drivers of *C9orf72*-related neuronal toxicity.^4–7^ We reported that micro-injection of poly(PR) encoding mRNA can cause an axonopathy in zebrafish embryos.^8^ Moreover, we observed that low dosages of DPRs caused axonal transport defects in human iPSC-derived motor neurons, similar to what was observed in neurons from *C9orf72* patient-derived iPSC-neurons.^9^ However, the mechanism(s) behind the DPR toxicity is not yet fully elucidated.

Genetic screens in simple experimental model organisms like yeast, flies, and worms have empowered the discovery of fundamental biological processes including the study of mechanisms of human disease.^10^ To identify genes in pathways responsible for a given disease phenotype, high throughput screens are very useful. With the advent of CRISPR/Cas9 genome editing, genome-wide genetic deletion screens have become possible using high complexity single-guide RNA (sgRNA) libraries.^11^ Two recent studies reported results from two genome wide pooled CRISPR/Cas9 screens that were performed in the K562 erythroid leukemia cell line or immortalized retinal pigment epithelial cells to investigate DPR toxicity.^11,12^ Candidate hits that either decreased cell death or decreased DPR production were subsequently confirmed in iPSC-derived neurons, murine neurons, and/or *Drosophila*.^11,12^ We hypothesized that an optimal CRISPR/Cas9 screen to identify modifiers of DPR toxicity should be done in disease-related neuronal cells derived from human, rather than the cell lines, not representing the CNS, used in the studies above.

As a number of kinases have been identified to play a key role in the pathogenesis of ALS/FTD and some of them are considered druggable targets,^13,14^ we performed a kinome-wide CRISPR/Cas9 screen in cortical neurons derived from iPSCs. The screen identified several candidate kinases, including NIMA-Related Kinase 6 (NEK6), involved in DPR-mediated toxicity. Specific NEK6 knock-down enhanced survival of poly(PR) treated neurons. We further demonstrated that knockdown or inhibition of NEK6 restored axonal transport defects in *C9orf72* patient-derived cortical neurons as well as the axonopathy in the poly(PR) zebrafish model. Furthermore, aberrant NEK6 expression in blood samples and brain tissues of *C9orf72* patients highlighted the important role of NEK6 in *C9orf72*-related toxicity. Finally, we demonstrated that knockdown and inhibition of NEK6 reversed DNA damage and reduced p53 activation observed in poly(PR) treated neurons or *C9orf72* iPSC-neurons. Therefore, we propose NEK6 as a novel therapeutic target for *C9orf72* ALS/FTD.

## 2 | METHODS

### 2.1 | CRISPR/Cas interference screen

The Brunello kinome-wide CRISPR/Cas9 KO library (3052 sgRNAs against 736 kinases, 4 sgRNA per gene) was purchased from Addgene (1000000082). The quality control of library and lentivirus packing was performed by Vector builder (VectorBuilder GmbH, Germany). About 8 million neuron progenitor cells (NPCs) at day in vitro (DIV) 46 NPCs per condition were transduced with the library at a Multiplicity of infection (MOI) = 0.3, and with a coverage of > 800 cells/sgRNA. Transduced cells underwent puromycin (1 *μ*g/ml) selection for 1 week. Cells were maintained in culture until DIV60, and then replated on poly-o-laminin coated plates at 50,000 cells/cm^2^. On DIV70, 50% of the wells were treated with Doxycycline (3 *μ*g/ml) with a daily medium change including Doxycycline until DIV77. At DIV79, PR20 (add 6 *μ*g/ml; Pepscan Presto BV, the Netherlands) was added to the culture medium to 50% of the culture. After 24 hours, culture wells were washed to remove the dead cells, and cells were harvested and frozen at −80°C or used in downstream assays. Genomic DNA was extracted from all conditions separately using the Midi Blood DNA extraction kit (51185, QIAGEN). The fragments containing sgRNAs were amplified by PCR and subsequently used for Next-Generation Sequencing (NGS) on an Illumina Nextseq platform to identify the barcodes of each sgRNA present. The bioinformatics tool MAGecK 0.5.724 was used for downstream analysis. First, sgRNAs were counted using “mageck count” directly from the sequencing fastq files. We then performed maximum-likelihood analysis of gene essentialities using “mageck mle” with the default parameters and using the library of 100 non-targeting guides as parameter for—control-sgRNA. (Full processing pipeline available on https://github.com/emc2cube/Bioinformatics). The enrichment of individual sgRNAs was calculated as log ratio, and gene level effects were calculated from 4 sgRNA targeting each gene. A *P*-value based confidence score was then derived as log-likelihood ratio describing the significance of the gene level effects. Gene ontology analysis was performed using the Gene Ontology Resource website.

### 2.2 | Axonal transport analysis

DIV79-DIV84 iPSC-derived cortical neurons were loaded with MitoTracker-Red (50 nM, Invitrogen), washed and left to equilibrate (20 minutes) in neuron maintenance medium (NMM), before transferring them to a HEPES buffered salt solution (pH 7.4, 150 mM NaCl, 5 mM KCl, 1 mM MgCl_2_, 2 mM CaCl_2_, 10 mM glucose, 10 mM HEPES). Measurements were performed on an inverted Zeiss Axiovert 200 M microscope (Carl Zeiss) with a 40× water immersion lens. Neurons were selected under differential interference optics (DIC) based on typical morphology consisting of a soma and long-extended neurites. MitoTracker-RED was excited at 580 nm, using a TILL Poly V light source (TILL Photonics) and image sequences were recorded (200 images at 1 Hz) onto a cooled CCD camera (PCO Sensicam-QE) using TillVisION (TILL Photonics) software. A heated gravity-fed perfusion system was used to keep cells at 36 ± 0.5°C during the recordings. All image analysis was performed in Igor Pro (Wavemetrics) using custom written routines based on a previously described analysis algorithm.^15^ In brief, kymographs or spatio-temporal maps were constructed for each of the neuronal processes. In these maps, stationary mitochondria appear as vertical lines and moving mitochondria generate tilted lines. Proportions of moving and stationary mitochondria were extracted from the maps by marking and analyzing the properties of each of the mitochondrial trajectories.

### 2.3 | Human *postmortem* brain tissue experiments

The recruitment protocol for collecting *postmortem* brain tissue (Table S4) was approved by the Human Ethics Committee at the University Hospital, Gasthuisberg, KU Leuven, Belgium. The tissue was collected in accordance with the applicable law in Belgium (UZ Leuven). At autopsy, the right hemisphere of the brain was dissected in coronal planes and frozen at −80°C. The left hemisphere was fixed in 4% phosphate-buffered formaldehyde. The *postmortem* diagnosis of ALS was pathologically confirmed by assessment of pTDP-43 pathology. For *C9orf72* repeat expansion determination, DNA was extracted from peripheral blood and/or cerebellum according to standard protocols. Analysis of the hexanucleotide repeat length in intron 1 of *C9orf72* was done by fragment length analysis by PCR and repeat-primed PCR (RP-PCR) as previously described.^16^ In addition, the presence of poly(GA) pathology was confirmed in the cortex.

For immunofluorescence evaluation, 5 *μ*m sections were cut from formalin-fixed paraffin-embedded tissue of the motor cortex. Slides were deparaffinized and epitopes were retrieved using low pH buffer. Afterward, a mix of anti-NEK6 plus anti-NeuN, anti-NEK6, plus anti-GFAP primary antibodies was applied overnight, followed by an antibody cocktail of species-specific Alexa488/Cy3/Cy5-conjugated secondary antibodies (Abcam and Jackson ImmunoResearch). Finally, the slides were mounted using ProLong Gold Antifade Mountant containing DAPI (Thermo Fisher Scientific) for counterstaining of the nuclei.

For Western blot studies, 50 mg of frozen motor cortex tissue was weighed and mechanically homogenized in 0.5 ml 2% SDS in TBS (Tris-buffered saline) with Nuclease (Pierce Universal Nuclease, Thermo Fisher Scientific) and a cocktail of protease/phosphatase inhibitors (Halt, Thermo Fisher Scientific) using a micropestle. Samples were sonicated, followed by a centrifugation at 14,000 *g* for 30 minutes. Afterward, the resultant supernatant was used for Western blotting. Protein concentrations were determined using the Pierce BCA Protein Assay Kit (Thermo Fisher Scientific).

### 2.4 | Zebrafish injections, SV2 immunohistochemistry, and phenotyping

Zebrafish work was performed as previously described.^8^ Zebrafish oocytes were injected at one-cell stage with the indicated amounts of the morpholinos. The splice-blocking morpholino against *Danio rerio nek6* (transcript ENSDART00000132599.2; morpholino sequence 5′-ATGTTAGAAAGTGTACCTCGATGCA-3′) and the standard control oligo (morpholino sequence 5′- CCTCTTACCTCAGTTACAATTTATA-3) were designed and generated by Gene Tools (Philomath, USA). Injected oocytes were incubated at 28°C. After 24 hours post fertilization (hpf) the embryos were dechorionated by using a forceps. Only morphologically normal embryos were selected for downstream experiments. At 30 hpf, the selected fish were deyolked and subsequently fixed overnight at 4°C in 4% formaldehyde in 1× PBS. Fish were permeabilized with acetone for 1 hour at −20°C, blocked with 1% BSA/1% DMSO/PBS for 1 hour at room temperature and immunostained with mouse anti-SV2 primary antibody (Table S2) followed by secondary antibody.

For phenotyping, 15 consecutive embryos per condition were analyzed with imaging (Leica DM 3000 LED microscope; DMK 33UX250 USB3.0 monochrome industrial camera, The Imaging Source, Bremen, Germany) and the Lucia software (version 4.60, Laboratory Imaging, Prague, Czech Republic) by a blinded observer. For the axonal length, a standardized method was used; five predefined and consecutive motor axons (i.e., the 8^th^ up to the 12^th^ axon on one side) were measured in all 15 embryos. Data for axonal length were normalized to the control condition. For the abnormal branching, a predefined set of 20 consecutive motor axons (i.e., the 8^th^ up to the 17^th^ axon on both sides) in the same 15 embryos were analyzed visually. Motor axons were considered abnormal when axons branched at or before the ventral edge of the notochord. An embryo was considered as having “abnormal branching” when at least two of these 20 axons were abnormal. For each experiment, a green fluorescent protein (GFP)-targeting morpholino was used as control at the same dose of the tested morpholino. Four biological replicates were performed. Axonal length and abnormal branching data represent mean ± 95% CI. Statistical analysis was done by one-way analysis of variance (ANOVA) and logistic regression.

### 2.5 | Quantification and statistics

A minimum of three independent experiments based on three biologically different differentiations were always performed. Statistical analysis was performed using Graphpad Prism version 5.0b. One-way ANOVA was used for the other experiments with post-hoc Tukey’s test to determine statistical differences between groups. *P<.05, **P<.01, ***P < .001, ****P < .0001 were considered significant. Data values represent mean ± SEM, unless indicated otherwise.

### 2.6 | Data availability

The data that support the findings of this study are available from the corresponding authors upon request. The RNA sequencing data have been deposited in the European Genome-phenome Archive (accession number EGAS00001004782).

### 2.7 | Additional methods

Other methods used in this study can be found in the Supplementary Methods online.

## 3 | RESULTS

### 3.1 | Kinome wide CRISPR/Cas9 screen in doxycycline-inducible Cas9-iPSC-derived cortical neurons identifies modifiers of poly(PR) toxicity

**FIGURE 1:**
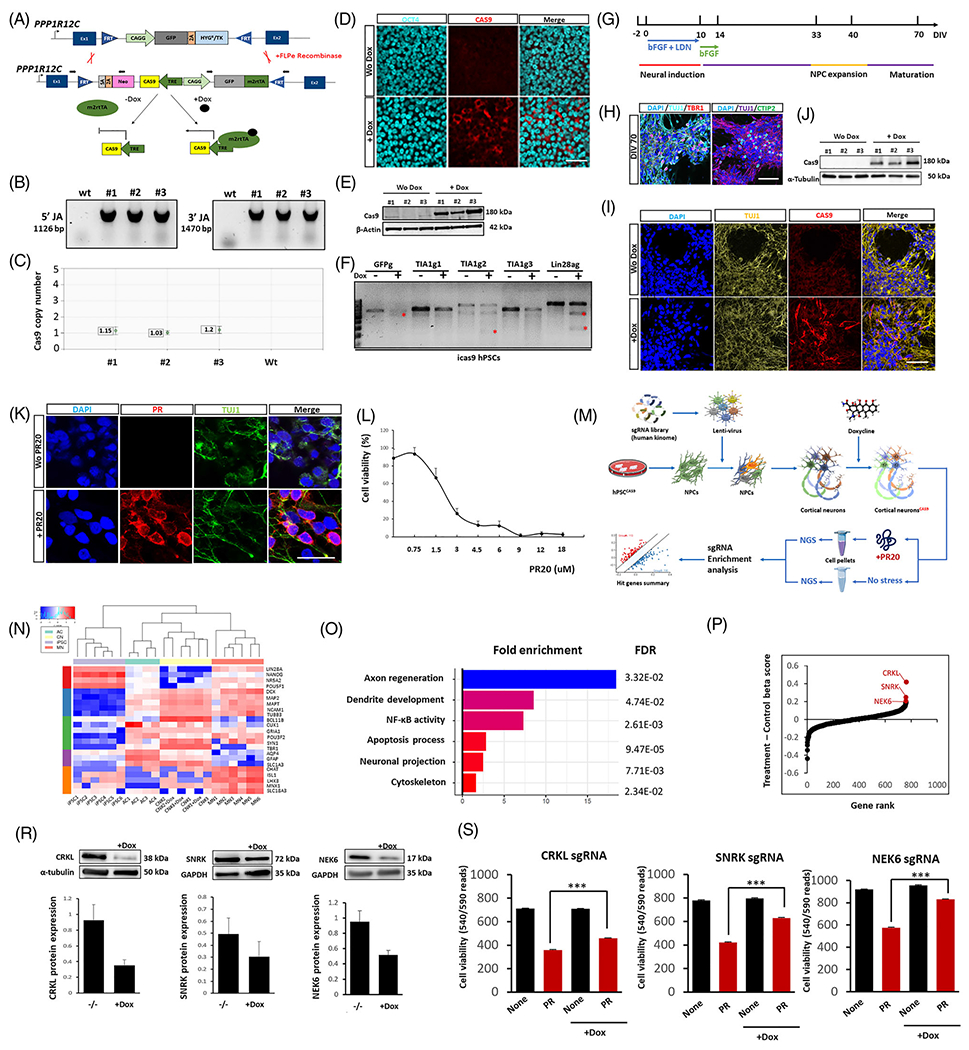
Kinome-wide CRISPR/Cas9 interference screen in doxycycline-inducible Cas9-iPSC-derived cortical neurons. (A) Schematic diagram of the strategy used to create inducible expression of *Cas9* in iPSC lines: schematic representation of the *AAVS1* locus, engineered to contain a flippase recognition target (FRTs)-flanked cassette suitable for recombinase-mediated cassette exchange (RMCE) using the pZ: F3-P TetOn-3×F-Cas9-F flanked by FRTs.^17^ This donor vector also contains a Tet-On system, including the M2 reverse tetracycline transactivator (M2rTA) and a tetracycline response element (TRE) upstream of a minimal cytomegalovirus (CMV) promoter. Neo: neomycin. (B) Identification of correctly targeted Cas9-iPSC cell clones by PCR, amplifying the 3′ and 5′ junctions (#1, #2, #3 denote the clone number, JA: Junction assay). (C) Digital droplet PCR (ddPCR) detection of copy number of *CAS9* in the BJ1 iPSC line. (D) Immunostaining validation of Cas9 expression after doxycycline (3 *μ*g/ml for 5 days) treatment in iPSCs. (E) Western blot validation of Cas9 expression after doxycycline treatment. (F) T7EI assay for Cas9-mediated cleavage in BJ1 Cas9-iPSCs using single sgRNAs targeting *GFP* (GFPg), T-cell-restricted intracellular antigen-1 (*TIA1*; TIA1g1, TIA1g2, TIA1g3), Lin-28 homolog A (*LIN28A*; LIN28Ag). (G) Schematic protocol of cortical neuron differentiation. LDN = LDN-193189. (H) Staining of DIV 80 PSC-cortical neuronal progeny for different cortical neuron markers (CTIP2 (or BCL11B) and T-box brain transcription factor 1 (TBR1)), neuron tubulin marker TUJ1 and DAPI. (I, J) Western blot and immunostaining for Cas9 expression in DIV80 PSC-neural progeny. (K) PR20 at a dosage of 6 *μ*M was visualized by immunocytochemistry in DIV80 neural progeny treated with or without PR20, Scale bar = 20 *μ*m. (L) PR20 dose-dependent cytotoxicity of DIV80 iPSC-neuronal progeny was measured by cell viability assay after a 24 hours treatment. (M) Schematic diagram of CRISPR/Cas9 screen procedure: DIV46 NPCs were transduced with the kinome-wide Brunello sgRNA vector library and transduced cells were selected with 1 *μ*g/ml puromycin; NPCs were differentiated to DIV70 cortical neurons, after which 3 *μ*g doxycycline was added for 5 days to induce Cas9, followed by treatment of 50% of the DIV 79 neuronal progeny with or without 6 *μ*M PR20. The surviving cells were subjected to deep sequencing and statistical analysis for sgRNA distribution. Five biological replicates were performed. (N) RNA sequencing was performed on DIV80 iCas9 cortical neurons not treated with PR20, collected in parallel with the screen. Shown is expression of genes typically found in astrocytes (AC), cortical neurons (CN), iPSC and motor neurons (MN) (control iPSC, AC, and MN obtained from public available data with Gene Expression Omnibus (GEO) accession number: GSE98290). (O) Functional enrichment of candidate modifiers (113 genes that upon KO enhance survival under PR20 treatment) identified from the screen based on Gene Ontology Resource. (P) Hit gene distribution with normalization by negative control sgRNAs. (R) Western blot for NEK6, SNRKor CRKL in DIV80 neurons (normalized to *α*-Tubulin or GAPDH) following transduction with single sgRNAs against *NEK6*, *SNRK*, or *CRKL*, respectively. (S) Cell viability of Cas9-iPSC-derived DIV47 neurons transduced with single sgRNA guide against *NEK6*, *SNRK*, or *CRKL* with and without 6 *μ*M PR20 treatment for 24 hours (n≥10). Data were plotted as mean ± SEM. Statistical significance was evaluated with one-way ANOVA and post-hoc Tukey’s test; *,**,*** *P* values of <0.05, <0.01, <0.001 respectively. Scale bar = 50 *μ*m

To enable a CRISPR/Cas9 screen in iPSC-derived cortical neurons, we created several inducible Cas9 (iCas9) expressing iPSC lines by inserting a single copy of the coding region of *Cas9* into the *AAVS1* locus under the control of a TET-On promoter system^17^ (Figure 1A and Figure S1A). As *Cas9* expression is doxycycline dependent, this enables induction of its expression at any stage during iPSC differentiation, here specifically in DIV70-85 iPSC-derived cortical neurons. As most sgRNA lentiviral libraries used for large screens contain a puromycin selectable cassette, we used a neomycin antibiotic resistance cassette to select for cells containing the i*Cas9* construct (Figure 1A). The correct insertion of the *Cas9* cassette was confirmed by a combination of a 3′ and 5′ junction assay polymerase chain reaction (PCR) and a digital droplet PCR (Figure 1B, C, Figure S1B). Induction of Cas9 expression was confirmed by Western blot and immunostaining (Figure 1D, E, Figure S1C). To prove the functionality of Cas9, iPSCs were transduced with individual sgRNAs, and a T7EI analysis was used to demonstrate cleavage of the target genes following induction of Cas9 (Figure 1F). Finally, cell identity, pluripotency, and genome integrity were confirmed by single nucleotide polymorphism (SNP) PCR, embryoid body Score Card analysis (Figure S1D, E), and array comparative genome hybridization (data not shown). By using a protocol adapted from Shi *et al*.,^18^ we demonstrated the differentiation of these iPSC lines into approximately 90% TUJ1 positive neurons on DIV70-85 (Figure S2A, B), containing a mixture of cells with layer VI and V cortical fate (Figure 1G, H). Following doxycycline administration, expression of Cas9 in cortical neurons was shown by qRT-PCR, Western blot, and immunostaining (Figure 1I, J, and Figure S1F). Therefore, these iCas9 iPSCs can be used for CRISPR/Cas9 screens in iPSC-derived cortical progeny.

We treated iCas9-iPSC-derived DIV79 neurons with PR20 peptides to mimic DPR toxicity.^11,12^ Poly(PR) was readily taken up and showed partial nuclear localization 24 hours after treatment (Figure 1K). A dose-response curve demonstrated that approximately 70%–80% iCas9-iPSC DIV80 neuronal progeny died when treated with 6 *μ*M PR20 (Figure 1L). This PR20 concentration was then used for the CRISPR/Cas9 live-death screen, following transduction with the Brunello human kinome-wide CRISPR/Cas9 KO pooled sgRNA library. iCas9 iPSCs were differentiated into NPCs, and NPCs were transduced with the lentiviral kinome sgRNA library at a MOI of 0.3. Transduced cells were selected by puromycin (Figure 1M). RNA sequencing performed on DIV80 cortical progeny from these transduced iCas9-NPCs demonstrated robust cortical neuronal differentiation (Figure 1N). sgRNA transduced NPCs were then differentiated into cortical neurons until DIV70, treated with doxycycline to induce Cas9 expression for 5 days, followed by PR20 treatment for 24 hours (Figure 1M). NGS was performed to identify sgRNAs enriched or depleted in the surviving PR20 treated cells ^19^ (Figure 1O and P). The screen identified 243 candidate genetic modifiers of PR20 toxicity, including 113 genes that enhanced and 130 that suppressed survival. Gene ontology analysis showed that the 113 genes protecting cells from PR20-mediated toxicity were involved in axon regeneration, dendrite development, apoptosis, and cytoskeletal organization (Figure 1O). To validate the results from the screen, we transduced iCas9-NPCs individually with sgRNAs directed against the top 3 candidate genes, *CRK like proto-oncogene*, *adaptor protein* (*CRKL*), *SNF-related kinase (SNRK)* and NIMA-related kinase 6 (*NEK6*), which enhanced survival in the interference screen (Figure 1P). A 50%–60% reduction in protein levels of CRKL, SNRK, and NEK6 was observed (Figure 1R, Figure S3), and an increased survival was noted in PR20 incubated cells transduced with sgRNAs against all three genes. sgRNAs against *NEK6* and *SNRK* displayed a more significant effect (Figure 1S).

### 3.2 | Knocking down NEK6 rescues axonal transport defects in iPSC-derived cortical neurons and the axonopathy in poly(PR) injected zebrafish embryos

Axonal transport defects are an early event in the pathogenesis of ALS.^9^ To assess whether axonal transport defects also occur in cortical neurons from healthy donor iPSC-derived cortical neurons treated with PR20 and from C9orf72 patient iPSC-derived cortical neurons, mitochondrial transport studies were performed. Consistent with our recent study,^9^ treatment of normal donor iPSC-DIV79 cortical neurons with PR20 significantly decreased axonal transport efficiency by nearly 50% (Figure S4A). To assess whether sgRNA-mediated knockdown of *CRKL*, *SNRK*, or *NEK6* reversed axonal transport defects caused by poly(PR), we transduced healthy donor iCas9-iPSC NPCs with individual sgRNAs targeting *CRKL*, *SNRK*, or *NEK6*. When DIV70 cortical neurons were treated with doxycycline and afterward with a non-lethal dose of PR20 (1.5 *μ*M), knocking down SNRK or NEK6, but not CRKL, rescued the axonal transport defect caused by PR20 in neurons (Figure S4A).

We next evaluated whether a similar functional rescue would also be found in neurons derived from iPSCs created from patients with C9orf72, containing spontaneous increased levels of DPRs. We treated DIV72 cortical neurons derived from iPSCs derived from one C9orf72 patient and its isogenic control, with antisense oligonucleotides (ASOs) targeting either *SNRK* or *NEK6*. Western blot demonstrated a clear decrease in SNRK or NEK6 levels in ASO-treated neurons (Figure S4B, C). As expected, axonal transport was also decreased with nearly 50% in iPSC-derived cortical neurons from the C9orf72 patient compared to its isogenic control (Figure S4C). We observed a full rescue of axonal transport defects in C9orf72 neurons by NEK6-specific but not SNRK-specific ASO treatment (Figure S4D). No effect was seen on axonal transport in isogenic control iPSC-neurons by NEK6-specific ASOs. With the fact that another NEK family member, NEK7, has been shown to be involved in microtubule stability,^20–22^ we further focused on the possible role of *NEK6* in neuronal defects caused by DPRs.

**FIGURE 2:**
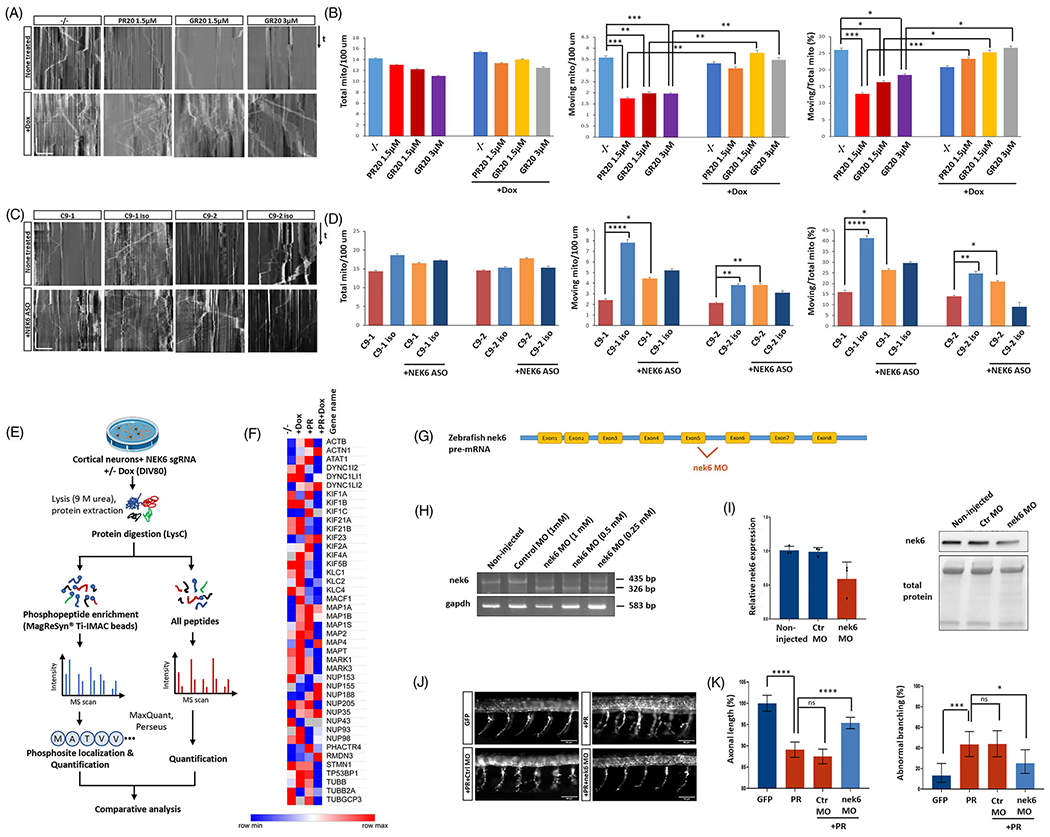
*NEK6* interference prevents poly(PR) toxicity in vitro and in vivo. (A) Representative kymographs of axonal transport recording in iCas9-iPSC-derived cortical neurons obtained from iCas9-iPSC-derived cortical neurons transduced with NEK6 sgRNA loaded with Mito-Tracker-Red (−/− = no treatment; +PR20 = PR20 treatment; +GR20 = GR20 treatment; +Dox = 3 *μ*g doxycycline treatment). (B) Quantification of total mitochondria, moving mitochondria and ratio of moving mitochondria to total mitochondria normalized to a neurite length of 100 *μ*m during 200 s in (A). (C) Representative kymographs of cortical neurons from two different C9orf72 patient iPSC as well as the respective isogenic control iPSC-derived neurons treated with or without different NEK6 ASOs for 1 week. (D) Quantification of total mitochondria, moving mitochondria and ratio of moving mitochondria to total mitochondria normalized to a neurite length of 100 *μ*m during 200 s in (C). (E) Schematic representation of the different steps to identify phosphorylation sites by mass spectrometry. The blue dots represent a phosphorylation site in proteins/peptides. The blue/red lines in the MS spectra represent site-determination. Interpretation of MS spectra involved both the identification of phosphopeptides and localization of the sites of phosphorylation. The final determination of phosphopeptides was calculated by referencing to the corresponding peptides from shot gun MS spectra. (F) Heatmap of identified phosphorylation sites in proteins involved in axonal transport and nucleocytoplasmic transport machineries. (G) The nek6 splice-blocking morpholino targets the exon5 intron5 splice junction of zebrafish nek6 pre-mRNA. (H) RT-PCR validation of morpholino-mediated nek6 modified splicing in zebrafish embryos at 30 hpf. Non-target morpholino at the highest concentration is used as standard control. Wild type nek6 is amplified as a 435 bp band, while aberrantly spliced nek6, induced by morpholino injection, is amplified as a 326 bp band. (I) Western blot validation of morpholino-mediated nek6 knockdown in zebrafish embryos at 6 hpf (normalized to Ponceau total protein staining). (J) Visualization of motor axons by immunohistochemistry (SV2 antibody) of 30 hours post fertilization (hpf) zebrafish embryos injected with 0.844 *μ*M GFP mRNA, 0.844 *μ*M poly(PR) mRNA, 0.844 *μ*M poly(PR) mRNA plus 0.5 mM control morpholino or 0.844 *μ*M poly(PR) mRNA plus 0.5 mM nek6 morpholino; scale bar = 50 *μ*m. (K) Quantification of axonal length (data represent mean ± 95% CI; one-way ANOVA) and aberrant axonal branching (data represent mean ± 95% CI; logistic regression) of zebrafish embryos in the four conditions; n = 4 biological replicates. All axonal transport measurements were performed on cortical neurons at DIV = 80-85; Number of neurons wherein axonal transport was measured ≥15 per data point; Kymographs scale bar time (vertical): 35 s; scale bar distance (horizontal): 25 *μ*m. Data were plotted as mean ± SEM. Statistical significance was evaluated with one-way ANOVA and post-hoc Tukey’s test; *, **, ***, ****P values < 0.05, <0.01, <0.001, and <0.0001, respectively

To address whether NEK6 knockdown also rescues axonal transport defects caused by other DPRs, we transduced healthy donor iCas9-hiPSC NPCs with *NEK6* sgRNA, treated DIV70 cortical neurons with doxycycline and afterward with a non-lethal dose of PR20 (1.5*μ*M), GR20 (1.5 *μ*M) and GR20 (3 *μ*M). Knockdown of NEK6 resulted in a full rescue of axonal transport defects caused by all three DPRs (Figure 2A, B). To further validate the rescue of axonal transport in cortical neurons from C9orf72 patient hiPSCs, we treated two different pairs of DIV72 C9orf72 patient-derived iPSC cortical neurons and their isogenic controls with antisense oligonucleotides (ASOs) targeting *NEK6* (Figure 2C, D). A significant rescue of axonal transport efficiency (40%–50%) was observed in both C9orf72 iPSC-cortical neurons treated with anti-*NEK6* ASOs, without any effects observed in the isogenic control neurons (Figure 2D). To gain insight into how loss of NEK6 may counteract neuronal death as well as axonal transport defects, we performed phosphoproteomics on *NEK6* sgRNA transduced iCas9-iPSC cortical neurons treated with or without doxycycline (Figure 2E). We identified significant changes in multiple phosphosites in the microtubule-associated protein 2 (MAP2) and microtubule-associated protein 1B (MAPB) protein following *NEK6* knockdown, as well as other proteins involved in axonal transport (Figure 2F).

Next, we wanted to confirm our in vitro data in an in vivo model. We performed a morpholino-mediated nek6 knockdown (Figure 2G, H and I) in a poly(PR) zebrafish model. We previously demonstrated that micro-injection of poly(PR) encoding mRNA resulted in a motor axonopathy in zebrafish embryos, comprising aberrant branching and reduced axonal length.^8^ We co-injected poly(PR) mRNA with a splice-blocking morpholino, specifically targeting the exon5-intron5 splice junction of the zebrafish *NEK6* orthologue, which is *nek6* (Figure 2G). Consistently, knockdown of *nek6* in zebrafish embryos injected with poly(PR), significantly ameliorated the axonal phenotype. More specifically, we observed a partial (± 30%–40%) normalization of axonal length and less aberrantly branched axons (Figure 2J, K).

### 3.3 | Dysregulation of NEK6 expression level in C9orf72 patient-derived PBMCs and *postmortem* brain tissues

**FIGURE 3:**
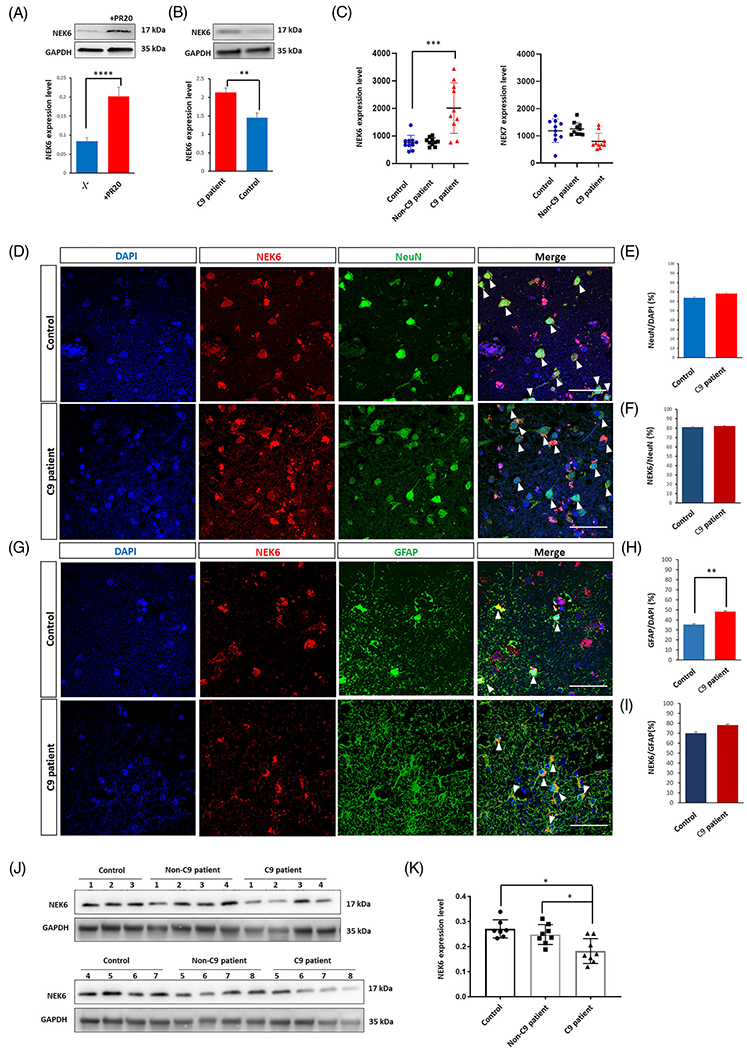
NEK6 expression profile in human iPSC-derived neurons and *C9orf72 postmortem* brain tissues. (A) Western blot analysis for NEK6 expression in iPSC-derived cortical neurons w/wo 1.5 *μ*M PR20 treatment. (B) Western blot analysis of NEK6 expression in *C9orf72* patient-derived cortical neurons and their isogenic controls. (C) qPCR detection of *NEK6 and NEK7* transcripts in PBMCs of healthy donors (control, n = 10), sporadic ALS patients without known mutations (Non-C9 patient, n = 10) and ALS patients carrying *C9orf72* mutation (*C9* patient, n = 10). (D) Immunostaining of NEK6 and NeuN in *postmortem* motor cortex tissues from *C9orf72* patients and non-neurological disorder case controls (white arrows indicate the colocalized NEK6 and NeuN staining). (E) Quantification of neuronal cell percentage in both conditions from (D). (F) Quantification of NEK6 expressing cell percentage in neuronal population from (D). (G) Immunostaining for NEK6 and GFAP in *postmortem* motor cortex tissues from *C9orf72* patients and non-neurological disorder case controls (white arrowheads indicate the colocalized NEK6 and GFAP staining). (H) Quantification of astrocyte percentage in both conditions from (G). (I) Quantification of NEK6 expressing cell percentage in astrocytes population from (G). (J) Western blot analysis of NEK6 expression in motor cortex tissue from ALS or FTD patients carrying *C9orf72* mutations (C9 patient, n = 8), non-neurological disorder case controls (Control, n = 7) and ALS or FTD patients that do not carry *C9orf72* mutations (Non-C9 patient, n = 8). (K) Quantification of (J). The images shown as a representative example were C9-1 case and Con-6 case. The quantification of the images was based on the C9-1 and C9-7 cases, and the Con-6 and Con-8 cases. For details about the human cases see Table S4. Data were plotted as mean ± SEM. Statistical significance was evaluated with one-way ANOVA and post-hoc Tukey’s test; *, **, *** *P* value of <0.05, <0.01, <0.001, respectively

*NEK6* belongs to the *Never In Mitosis A-like kinase* (*NEK*) gene family.^23^ Within the family, NEK7 is very closely related to NEK6 in structure as well as in function.^23^ We investigated the expression level of NEK6 in human-derived neurons and tissues. Western blot demonstrated a significant increase in NEK6 levels in iPSC-derived cortical neurons treated with PR20 compared to untreated control neurons (Figure 3A, Figure S5), as well as in C9orf72 iPSC-derived cortical neurons compared to their isogenic controls (Figure 3B, Figure S6). In addition, we observed a significant upregulation of *NEK6* but not *NEK7* transcripts in peripheral blood mononuclear cells (PBMCs) from 10 ALS patients carrying C9orf72 repeat expansions compared to 10 healthy controls and 10 sporadic ALS patients (Table S3, Figure 3C). Thus, NEK6, but not NEK7, appeared to be dysregulated in C9orf72 cells and tissues. To investigate NEK6 expression levels in end-stage disease, we evaluated NEK6 levels in *postmortem* motor cortex from C9orf72 patients, healthy controls and non-C9orf72 ALS/FTD patients (Table S4). We first assessed the presence of NeuN-positive neurons versus GFAP-positive astrocytes in these *postmortem* brain tissues. This demonstrated similar percentages of neurons (Figure 3D, E) in brain slices from C9orf72 patients and healthy controls. In line with the known astrogliosis in ALS,^24^ C9orf72 brain tissue contained increased numbers of astrocytes (Figure 3G, H) compared with healthy control brain tissue. NEK6 was located mainly in the neuronal cells in both groups (Figure 3F), but was also present in astrocytes where slightly more patient astrocytes stained positive for NEK6 than healthy control astrocytes (Figure 3I). Next, we performed Western blot to quantify the NEK6 protein level in these tissues and found significantly lower NEK6 protein levels in C9orf72 brain tissue (Figure 3J, K, Figure S7), which might suggest a protective effect from down regulated NEK6 levels in the remaining neuronal cells in end stage brain tissue.

### 3.4 | Inhibiting NEK6 rescues p53-related DNA damage caused by C9orf72 mutation and poly(PR) toxicity

**FIGURE 4:**
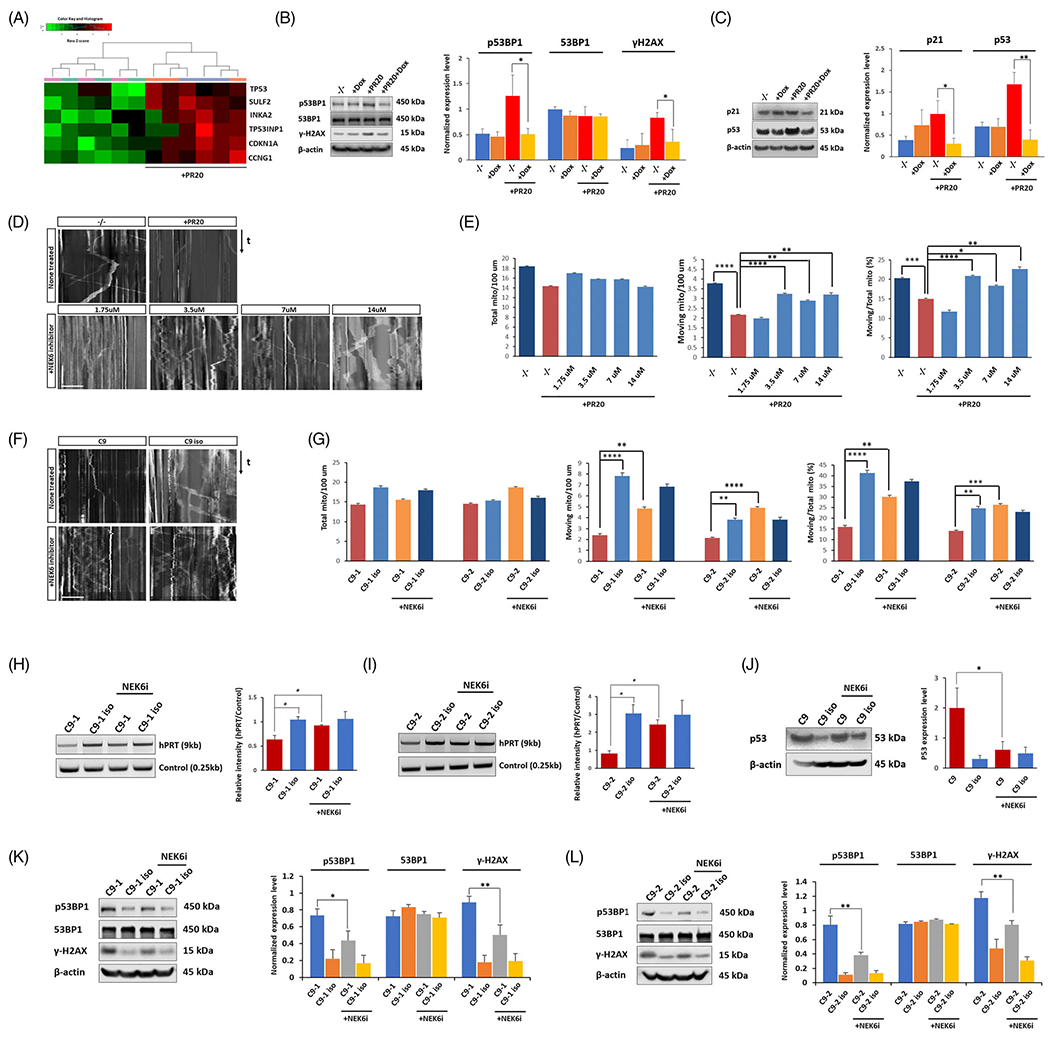
Inhibiting NEK6 counteracts p53 pathway activation, DNA damage and axonal transport defects caused by DPRs/*C9orf72* mutation. (A) Heat map for p53 pathway-related gene expression from RNA sequencing of DIV80 iCas9 cortical neurons w/wo PR20 treatment. (B) Western blot analysis of DNA damage marker p53BP1 and *γ*H2AX in iCas9-iPSC-derived cortical neurons obtained from iCas9-iPSC-derived cortical neurons transduced with NEK6 sgRNA w/wo PR20 treatment. (C) Western blot analysis for p53-related senescence gene expression (p53 and p21) in all conditions from (B). (D) Representative kymographs of axonal transport recordings in iPSC-derived cortical neurons treated with PR20 peptides and different concentrations of the NEK6 inhibitor, inhibitor 8 (DC12465, DC chemicals, China) starting 3 days before adding PR20. (E) Quantification of total mitochondria, moving mitochondria and ratio of moving mitochondria to total mitochondria normalized to a neurite length of 100 *μ*m during 200 s in (D). (F) Representative kymographs of axonal transport recording in *C9orf72* patient and their isogenic control-derived cortical neurons w/wo NEK6 inhibitor 8 treatment (3.5 *μ*M, 3 days). (G) Quantification of total mitochondria, moving mitochondria and ratio of moving mitochondria to total mitochondria normalized to a neurite length of 100 *μ*m during 200 s in (F). (H) LA-PCR of hPRT genes in *C9orf72* patient 1 and its isogenic control-derived cortical neurons w/wo NEK6 inhibitor 8 treatment. (I) LA-PCR of hPRT genes in *C9orf72* patient 2 and its isogenic control-derived cortical neurons w/wo NEK6 inhibitor 8 treatment. (J) Western blot analysis of p53 expression in (I). (K) Western blot analysis of DNA damage marker p53BP1 and *γ*H2AX in neurons from iPSC derived from C9orf72 patient 1 and its isogenic control-derived cortical neurons following NEK6 inhibitor treatment. (L) Western blot analysis of DNA damage marker p53BP1 and *γ*H2AX in neurons from iPSC derived from C9orf72 patient 2 and its isogenic control-derived cortical neurons following NEK6 inhibitor treatment. Data were plotted as mean ± SEM. Statistical significance was evaluated with one-way ANOVA and post-hoc Tukey’s test; *,**,***, **** *P* value of <0.05, <0.01, <0.001, <0.0001, respectively

Based on the phosphoproteomics data, we also observed a significant decrease in phosphorylation of the p53 binding protein 1 (p53BP1) following knockdown of *NEK6* in PR20 treated neurons (Figure 2F). It has been shown that 53BP1 is a crucial regulator of the p53-related DNA damage response pathway and C9orf72 repeat expansion can induce DNA damage in patient tissues.^25,26^ Phosphorylated 53BP1 (p53BP1) protein plays an active role in DNA double strand break repair.^25^ Bulk RNA seq of iPSC-derived cortical neurons with or without PR20 treatment showed an upregulation of multiple p53 pathway related senescence markers in the PR20 treated condition (Figure 4A). Transcriptional analysis based on different published datasets^27,28^ also identified a consistent upregulation of the cyclin dependent kinase inhibitor 1A (*CDKN1A*) gene (coding for the p21 protein, a crucial downstream senescence marker in the p53-related DNA damage pathway) in C9orf72 patient iPSC-derived neurons (Figure S8A, B). Western blot demonstrated significantly higher levels of the DNA damage markers p53BP1 and *γ*H2AX in iPSC-derived cortical neurons treated with PR20, and knockdown of *NEK6* reversed the expression level of both back to normal (Figure 4B). Therefore, decreased phosphorylation of p53BP1 and *γ*H2AX expression is consistent with decreased p53-related DNA damage following *NEK6* knockdown in poly(PR) treated neurons. Indeed, knockdown of *NEK6* also significantly decreased the upregulation of p53 and p21 in iPSC-derived cortical neurons treated with PR20 (Figure 4C).

Finally, we treated iCas9-iPSC-derived cortical neurons 3 days before PR20 treatment with a recently described NEK6 inhibitor, Inhibitor 8.^29^ Treatment with this inhibitor, already at a concentration of 3.5 *μ*M, increased the axonal transport efficiency with nearly 40% in cortical neurons treated with PR20 (Figure 4D, E). When we treated the two pairs of C9orf72 patient iPSC-derived cortical neurons and their isogenic controls with NEK6 inhibitor 8, we also observed a full rescue of the axonal transport defect in C9orf72 iPSC-neurons, while no effects were observed in the isogenic controls (Figure 4F, G). Long amplicon polymerase chain reaction (LA-PCR) further demonstrated the presence of increased DNA damage in in C9orf72 patient iPSC-derived cortical neurons compared to isogenic control cells (Figure 4H, I). The NEK6 inhibitor 8 also rescued DNA damage in C9orf72 patient-derived cortical neurons without affecting the isogenic controls (Figure 4H, I). The significantly upregulated levels of p53 in C9orf72 patient-derived cortical neurons, as demonstrated by Western blot, were also reversed by NEK6 inhibitor 8 treatment (Figure 4J). Furthermore, we assessed p53BP1, 53BP1, and *γ*H2AX levels in the two pairs of C9orf72 patient and their isogenic control-derived cortical neurons with and without NEK6 inhibitor 8 treatment (Figure 4K, L). A significant decrease of p53BP1 and *γ*H2AX was observed in C9orf72 patient-derived cortical neurons after NEK6 inhibitor 8 treatment, while no effect was seen on isogenic control-derived cortical neurons (Figure 4K, J). This result further confirmed that NEK6 inhibition rescues p53-related DNA damage in C9orf72 patient-derived cortical neurons.

## 4 | DISCUSSION

In this study, we describe the first pooled CRISPR/Cas9 screen in iPSC-derived cortical neurons, modeling poly(PR) toxicity caused by mutations in *C9orf72*. We identified *NEK6* as a novel disease modifier, as decreased *NEK6* expression rescued iPSC-derived cortical neuron cell death caused by high concentrations of poly(PR20), and rescued defects in axonal transport caused by low concentrations of poly(PR). Furthermore, knockdown of *NEK6* in *C9orf72* patient-derived iPSC-cortical neurons also rescued axonal transport defects. Consistently, morpholino-mediated knockdown of *nek6* in zebrafish embryos, also significantly rescued the poly(PR)-induced motor axonopathy, validating the role of NEK6 in the DPR-mediated axonopathy in vivo. We also found that NEK6 expression is dysregulated in blood cells and *postmortem* brain tissues of *C9orf72* patients, further substantiating an important role for NEK6 in C9orf72-related neurodegeneration and identifying a potential new biomarker for *C9orf72* FTD/ALS. At the mechanistic level, we demonstrated that knockdown and pharmacological inhibition of NEK6 rescued DNA damage and p53 pathway upregulation in both poly(PR) treated or *C9orf72* patient-derived cortical neurons. Therefore, our data strongly indicate that targeting *NEK6* might be a new therapeutic strategy counteracting the toxic effects of DPRs in *C9orf72* FTD/ALS.

*NEK6* is a member of the *NEK* family of kinases that generally affect the mitotic process.^23^ NEK6 clusters together with two other NEK kinases, NEK7 and NEK9.^30^ Interestingly, NEK7, which has 87% amino acid identity in the predicted kinase domain with NEK6,^31^ was also among the top 20 kinases identified in our screen, that when decreased, rescued neurons from PR20 toxicity. Together with NEK7, NEK6 has been reported to participate in the establishment of the microtubule based mitotic spindle, and is involved in G2/M phase cell cycle arrest induced by DNA damage.^32^ Recent studies also reported that NEK7 plays a role in neuronal survival and microtubule dynamics.^20,21^ This is in line with our phosphoproteomics studies demonstrating that knockdown of NEK6 affected the phosphor sites in proteins that are part of microtubules, such as MAP2 and MAPB1. Furthermore, NEK6 has known or predicted interactions not only with NEK7 and NEK9, but also with a number of nucleoporins (NUPs), the major building blocks of nuclear pore complexes.^33^ Nuclear pores are the gateways connecting the nucleoplasm and cytoplasm,^34^ and disruption in nuclear import-export has been proposed to play an important role in neurodegeneration.^34^ A genome-wide CRISPR/Cas9 screen in K562 cells identified several nuclear import/export proteins (NXT1, XPO6, IPO11, and NPIPB6) as modifiers of DPR toxicity.^11^ Another CRISPR/Cas9 screen in retinal pigment epithelial cells transduced with *C9orf72* repeat expansion constructs identified genes involved in RNA metabolism and transport as potential disease modifiers of DPR toxicity,^12^ where RNA transport processes are highly dependent on the nuclear transport machinery.^12,35^ Hence, our screen identified similar processes involved in DPR toxicity and DPR-mediated axonal transport defects as described in these two studies wherein a CRISPR/Cas9 screen was done in non-disease relevant cells.

Recent studies identified mutations in another *NEK* family member, *NEK1*, as a risk factor for both sALS^36^ and fALS where *NEK1* variants occur together with other ALS genes, including *superoxide dismutase 1* (*SOD1*), *C9orf72*, *TAR DNA-binding protein 43* (*TARDBP)*, and *tubulin beta 4A Class IVa* (*TUBB4A)*.^37,38^ Most *NEK1* variants cause loss of function.^37^ Although, the exact mechanism underling NEK1 causing ALS is still unclear, an interaction between NEK1 and another ALS gene, *chromosome 21 open reading frame 2 gene* (*C21orf2)*, has been reported as being important to ensure an efficient DNA damage repair response.^39^ In contrast, we demonstrated that loss of NEK6 may be beneficial for *C9orf72* neurons or neurons exposed to DPRs. NEK1 and NEK6 are very different, both in protein structure and disease involvement.^23^ Here, we demonstrate for the first time that NEK6 may be involved in neurodegeneration.^40^ We observed that NEK6 is expressed higher in iPSC-derived cortical neurons treated with poly(PR), in cortical neurons from C9orf72 patient iPSCs as well as in PBMCs from C9orf72 patients. In contrast, we found that NEK6 protein levels were lower in *postmortem* cortical tissue from C9orf27 ALS/FTD patients. As iPSC-derived neurons most likely mimic early stages of the disease and as the PBMC samples were obtained from patients at diagnosis, that is, a comparably early stage of disease, it is possible that elevated levels of NEK6 in PBMC may serve as an early biomarker for C9orf72 disease, while the lower levels of NEK6 found in end stage *postmortem* tissues may suggest that low levels of NEK6 may have a protective role in the remaining cells, just like in our CRISPR/Cas9 screen. Indeed, the observation that treatment with the NEK6 inhibitor, inhibitor 8, rescued axonal transport defects in iPSC-derived cortical neurons both following DPR treatment and from C9orf72 patients, supports the notion that downregulating NEK6 levels/activity has a protective effect, in contrast to the harmful effects of decreased NEK1 levels shown in ALS.^37^ Therefore, NEK6 may play a crucial regulatory role in C9orf72-related toxicity.

Recently, p53 was shown to be a central regulator of axonal degeneration caused by poly(PR) toxicity, as these DPRs induce p53 stabilization (phospho-P53) and enhance transcription of p53 target genes.^41^ In line with that study, our RNA seq and phosphoproteomics data of PR20 exposed neurons identified activation of p53-related senescence and DNA damage response genes. Walkers et al., demonstrated that there is a significant accumulation of genome damage and induction of cellular senescence in C9orf72-models.^42^ Other studies also demonstrated increased DNA damage levels, which cause axonal degeneration or axonal transport defects in different ALS iPSC models.^41,43^ In general, genome damage-mediated cellular senescence is p53-mediated. It is thus possible that extensive genome damage in C9orf72 cellular models causes p53 dependent senescence, which may be the primary pathway of neurotoxicity, including axonal transport defects. There are also studies that link NEK family genes to p53-dependent cellular senescence.^44,45^ We found that NEK6 elimination/inhibition rescued DNA damage and decreased the expression level of p53 pathway-related genes caused by either poly(PR) treatment or the C9orf72 mutations. While cellular senescence involves cell cycle arrest in cycling cells like astrocytes or neural progenitor cells, several recent studies suggest that neurons also undergo senescence, which is characterized by a dormant state with reduced transcription and neuronal activity to prevent stress-induced neuronal cell cycle re-entry.^46,47^ Therefore, inhibition of NEK6, and as a consequence blocking activation of the DNA damage-related p53 pathway, may provide a window for neuronal repair. Nevertheless, the exact mechanism by which NEK6 regulates this process remains to be further clarified. One possibility would be that NEK6 is involved in the DNA damage response (DDR) through regulating posttranslational modification of DDR factors (e.g., phosphorylation of 53BP1 and *γ*H2AX) and, hereby, affects the p53 signaling transduction pathway cascade including the downstream factor p21, as we show here, or PUMA, as shown by Maor-Nof et al.,^41^ eventually causing axonal degeneration and apoptosis. Another possibility is that NEK6 directly interacts with DPRs to trigger senescence. The N-terminal transactivation domain (TAD) of p53 can directly interact with poly(PR/GR), in a process which is regulated through liquid-liquid phase separation (LLPS) of p53.^48^ p53 TAD has also been reported to bind to CBP/p300 (cAMP-response-element-binding protein [CREB]-binding protein) and CBP/p300 stabilizes p53 in response to DNA damage by preventing MDM2-mediated p53 degradation.^49,50^ Therefore, high levels of NEK6 may regulate the interaction of p53 TAD and CBP/p300 during DNA damage caused by poly(PR/GR) and cause increased p53 stability and lead to axon degeneration.

We also demonstrated that pharmacological inhibition of NEK6 can successfully rescue axonal transport defects, DNA damage and decrease p53 expression in both poly(PR) treated iPSC-derived cortical neurons and C9orf72 patient iPSC-derived cortical neurons. These data provide confirmation that NEK6 inhibition modulates the stress induced DNA damage response by inhibiting p53, resulting in a block of DNA damage induced cellular senescence. More importantly, our study emphasizes that the kinase function of NEK6 plays a pivotal role in regulating C9orf72 repeat expansion induced neurotoxicity via the p53 pathway and hence might be a novel promising drug target that can interfere with p53-related DNA damage caused by DPRs in C9orf72. We hypothesize that NEK6 may play a role upstream from p53 in the DNA damage pathway activated by poly(PR) stress, while PUMA (proposed by M Maor-Nof et al.^41^ as a target for p53 pathway) may play a role downstream of p53 in cell apoptosis processes in general. Thus, NEK6 may be a more attractive drug target. Therefore, it will be of interest to develop additional and more specific inhibitors of NEK6 kinase activity that can also cross the blood brain barrier and test their potential application in treating C9orf72-related ALS/FTD.

## Supplementary Material

supinfo

## RESEARCH IN CONTEXT

1. **Systematic Review:** The most important genetic cause of the neurodegeneration leading to frontotemporal dementia (FTD) or amyotrophic lateral sclerosis (ALS) are hexanucleotide repeats in *C9orf72*. 1. These repeats give rise to dipeptide repeat proteins, the most toxic ones being those containing arginines (e.g., poly(PR)). 2. Therefore, finding new modifiers to reverse poly(PR) toxicity is vital important to provide new therapeutic approaches for *C9orf72*-related FTD or ALS.
2. **Interpretation:** Our study discovered, validated, and explored the therapeutic potential of the kinase, NEK6, to block poly(PR) toxicity from the *C9orf72* repeat expansion. We demonstrated a new role of NEK6 in FTD/ALS and the beneficial effect of inhibiting NEK6 to reverse p53-mediated DNA damage and neuronal toxicity that was caused by the *C9orf72* repeats expansion.
3. **Future Directions:** It will be important to further understand the complete mechanism(s) of NEK6 in *C9orf72*-related pathological conditions. In addition, it will be important to develop reliable NEK6 inhibitors that are more specific and have minimal side effects, suitable to treat *C9orf72* patients.
