# Supplementary material for: CRISPR/Cas9 screen in human iPSC-derived cortical neurons identifies NEK6 as a novel disease modifier of C9orf72 poly(PR) toxicity
Source: Alzheimers Dement. Author manuscript; Available in PMC 2024 Apr 1. (PMC9943798; doi:10.1002/alz.12760)
Supplement: supinfo [file NIHMS1825305-supplement-supinfo.docx]

**Supplementary Figures:**

**
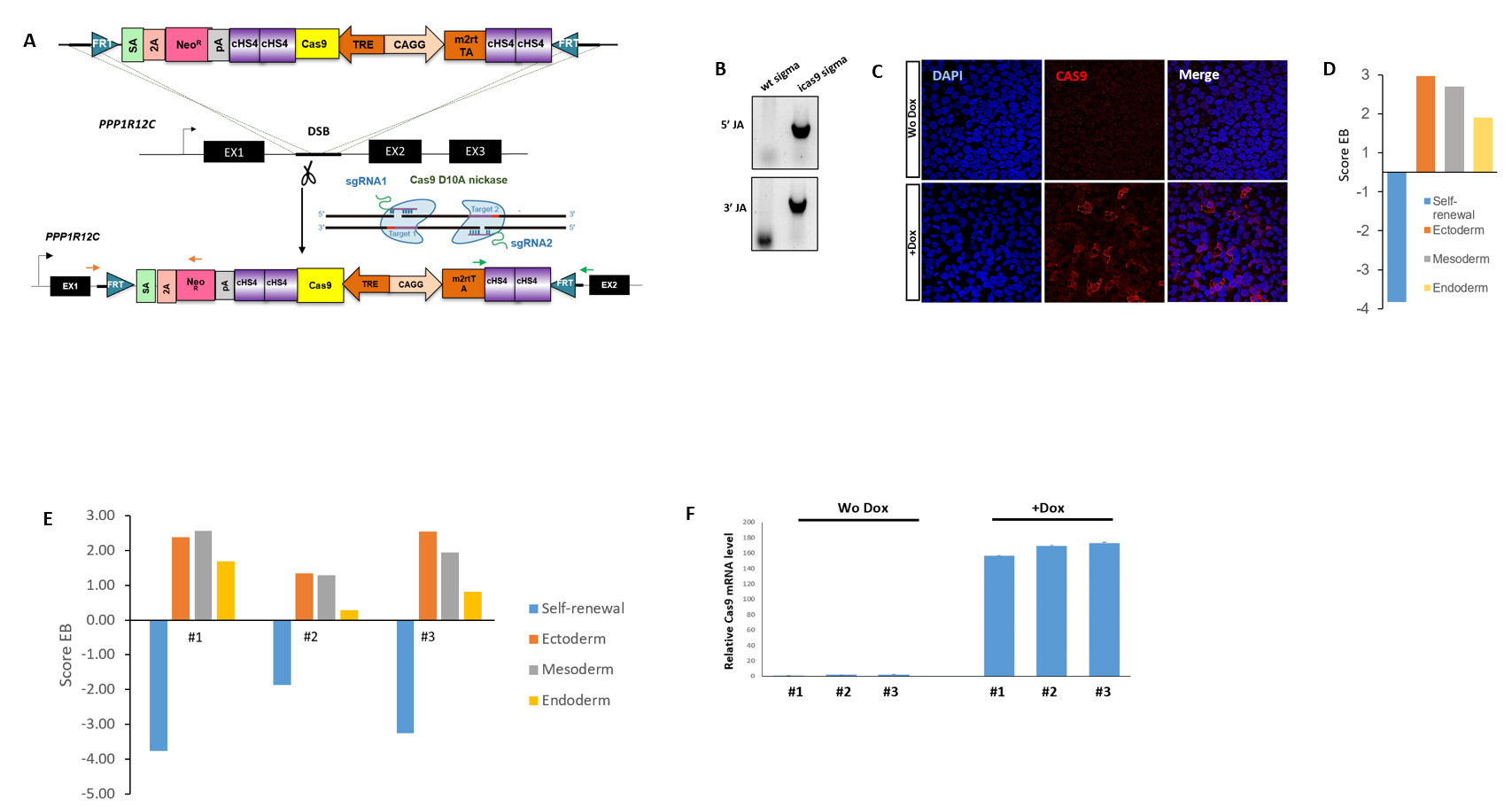
**

**Supplementary Figure 1. Characterization of iCas9 iPSC lines**

(A) Schematic diagram of the strategy used to create an inducible overexpressing Cas9 IPSC00028 by CRISPR/nickase mediated-homology recombination in AAVS1 locus using the donor plasmid containing TetOn controlled Cas9 in Sigma background; (B) Identification of positive Cas9-iPSC cell clones by PCR amplification with primers amplifying 3’ and 5’ junctions of Sigma Cas9-iPSC; (C) Immunostaining validation of Cas9 expression after doxycycline treatment in Sigma Cas9-iPSC; (D) Embryoid body formation followed by Score card® analysis to demonstrate pluripotency, which is presented by gene markers from the three germ layers of Sigma Cas9-iPSC; (E) Embryoid body formation followed by Score card® analysis to demonstrate pluripotency, which is presented by gene markers from the three germ layers of BJ1 Cas9-iPSC line; (F) qPCR detection of Cas9 expression at mRNA level of BJ1 Cas9-iPSC-derived cortical neurons (DIV80) following treatment with doxycycline.

**
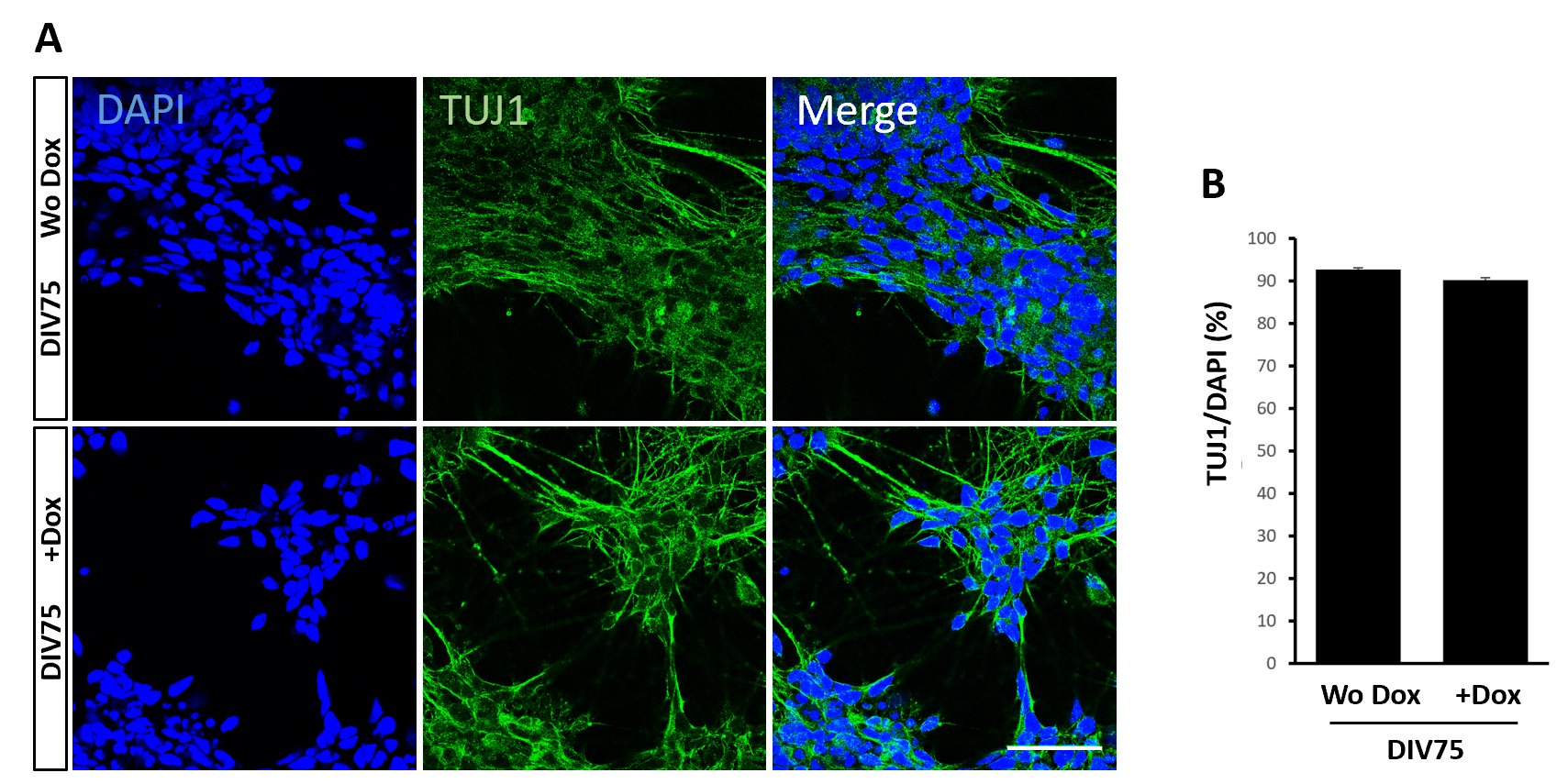
**

**Supplementary Figure 2.** **Neuronal differentiation of inducible Cas9 hiPSC**

(A) Immunostaining validation of neuronal differentiation of iCas9 hiPSC at DIV75 with and without Doxycycline treatment; (B) Quantification of percentages of neuronal cells (TUJ1 positive) in both conditions.

**
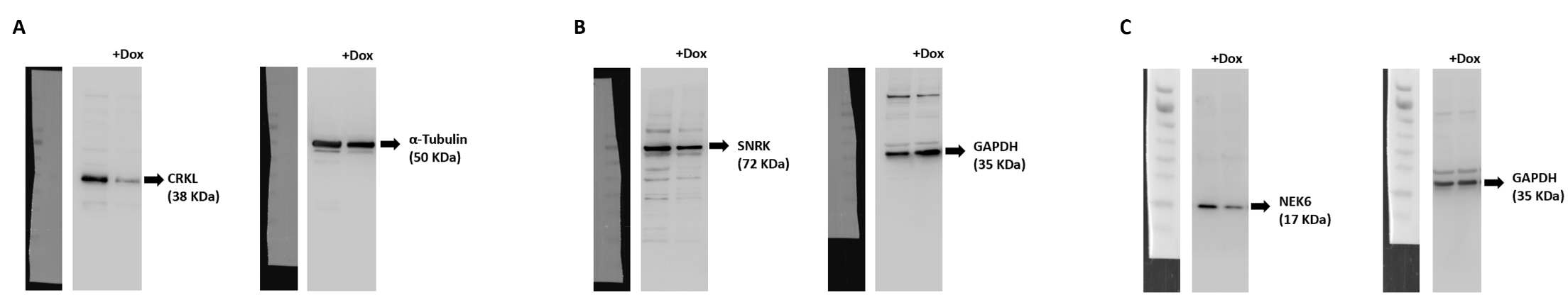
**

**Supplementary Figure 3.** **Full western blot for NEK6, SNRK or CRKL in DIV80 neurons**

**
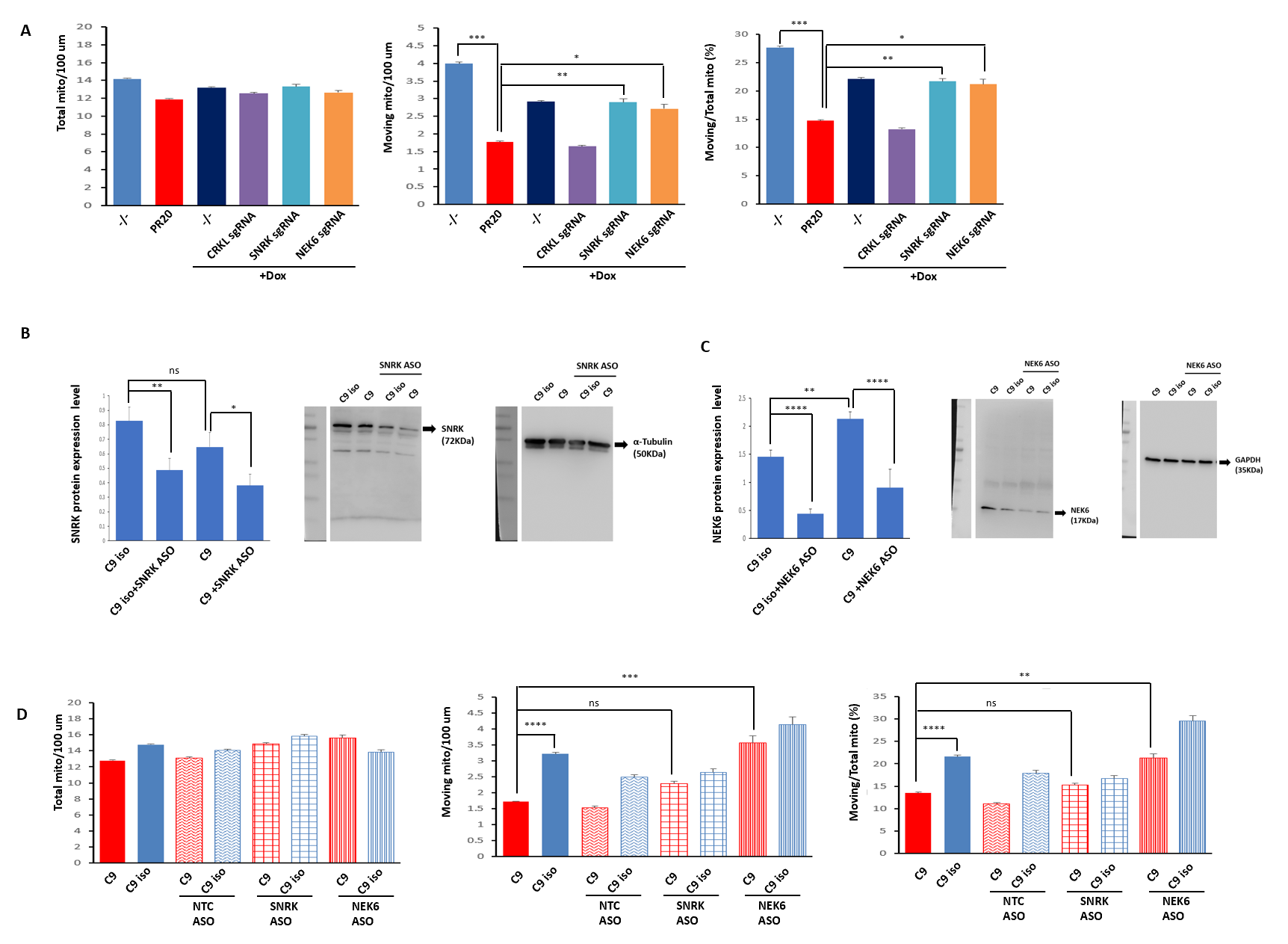
**

**Supplementary Figure 4.** **Axonal transport studies DPR treated or *C9orf72* cortical neurons with and without kinase interference**

(A) Quantiﬁcation of total, moving mitochondria and ratio of moving to total mitochondria normalized to a neurite length of 100 µm during 200 s in Cas9-iPSC-derived cortical neurons transduced with CRKL sgRNA, SNRK sgRNA and NEK6 sgRNA with PR20 treatment; (B) Western blot validation of ASO-mediated knockdown of SNRK in iPSC-derived cortical neurons; (C) Western blot validation of ASO-mediated knockdown of NEK6 in iPSC-derived cortical neurons; (D) Quantiﬁcation of total, moving mitochondria and ratio of moving to total mitochondria in iPSC-derived cortical neurons from isogenic control and *C9orf72* ALS/FTD patient treated with different NTC ASO, SNRK ASO and NEK6 ASO. Axonal transport measurements were done in >15 number of neurons per data point. One-way ANOVA with post-hoc Tukey’s test, Data values represent mean ± SEM

**
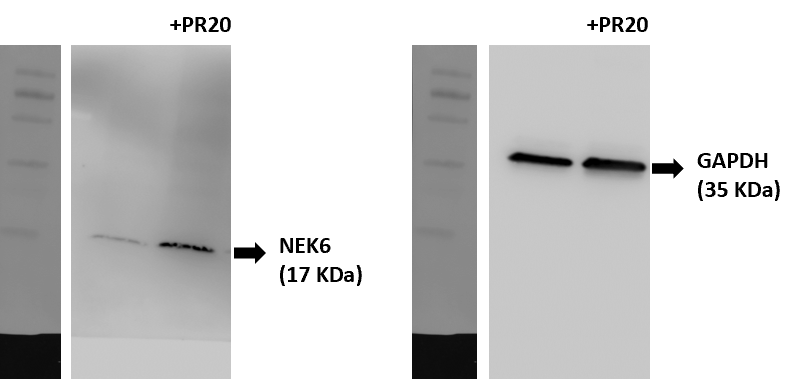
**

**Supplementary Figure 5. Full western blot of NEK6 in hiPSC derived cortical neurons w/wo PR20 treatment**

**
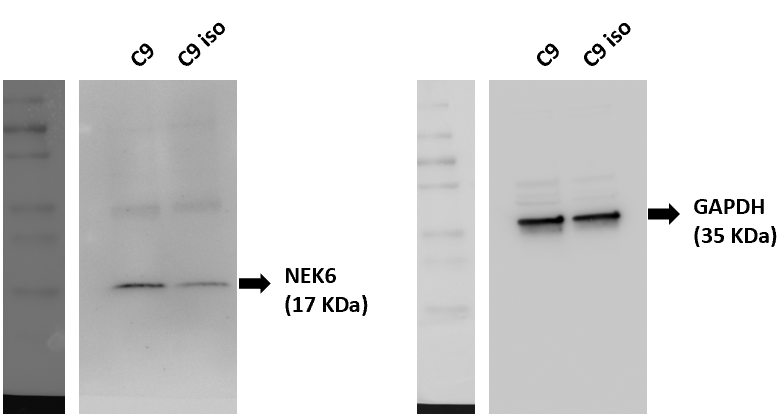
**

**Supplementary Figure 6. Full western blot of NEK6 in C9orf72 patient and its isogenic control derived cortical neurons**

**
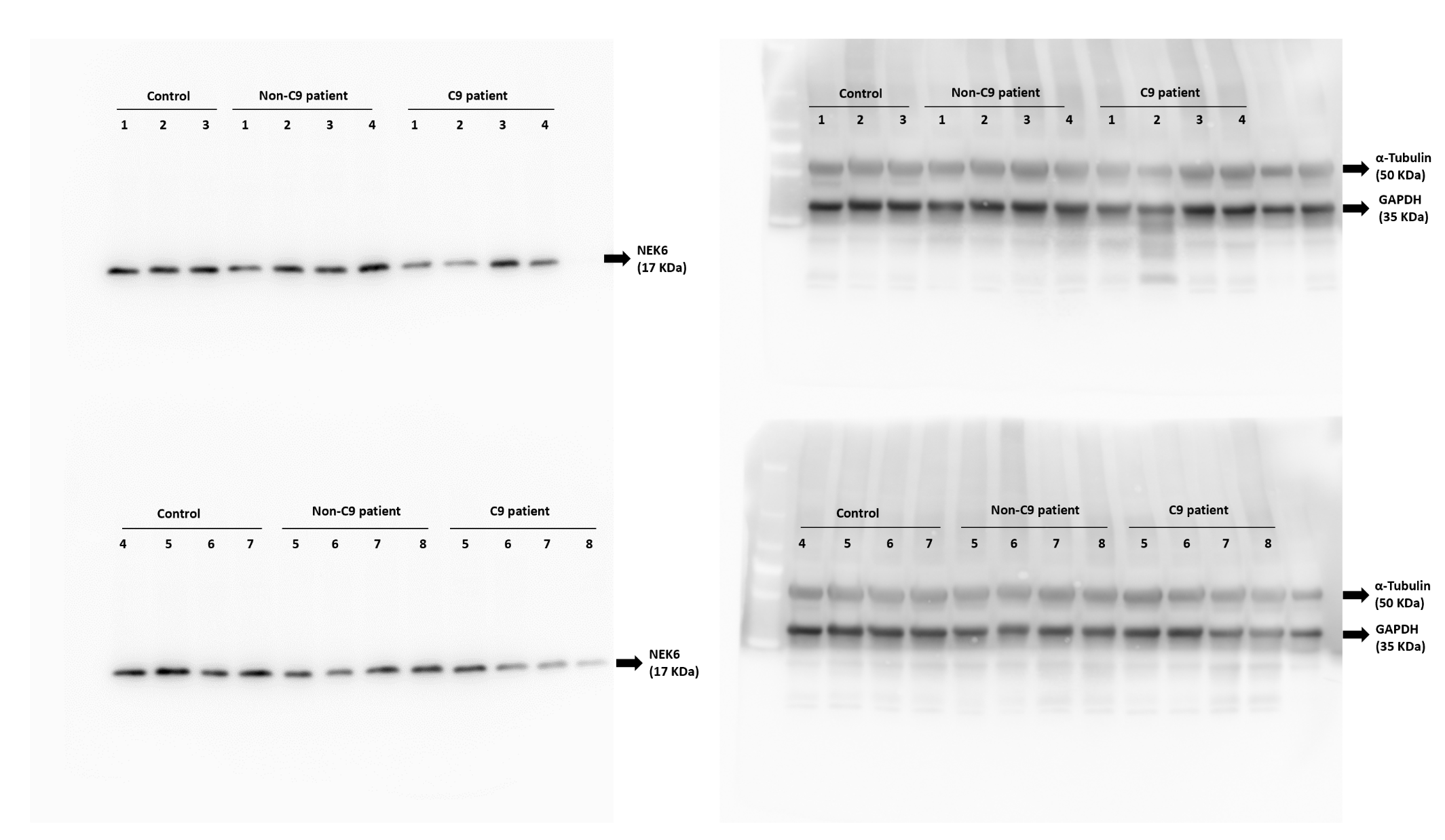
**

**Supplementary Figure 7. Full western blot of NEK6 in postmortem brain tissues**


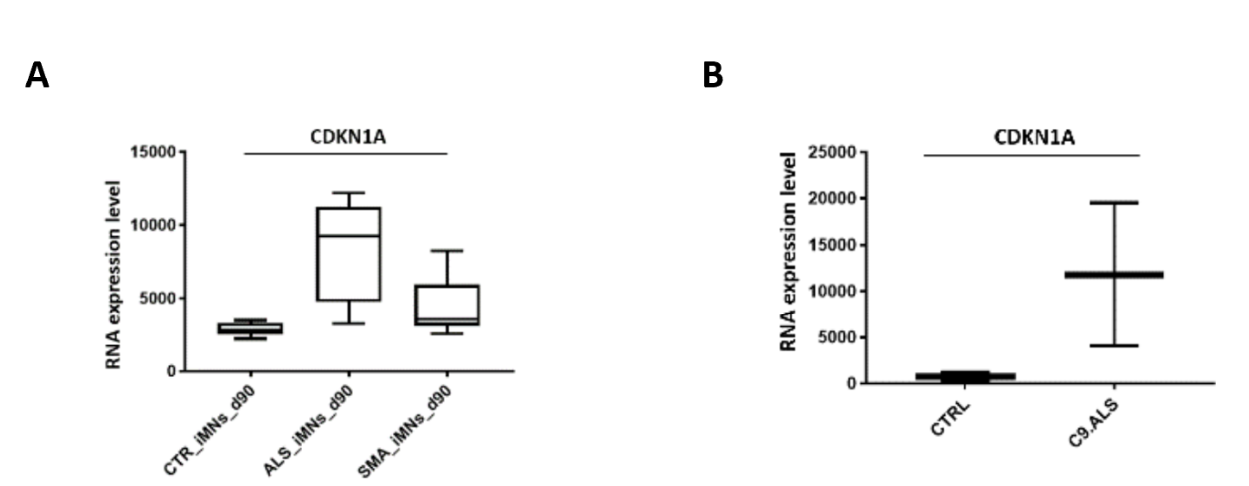


**Supplementary Figure 8. p53 pathway marker expression**

(A) RNA expression level of *CDKN1A* in iPSC-derived neurons on DIV90 of differentiation from healthy control, *C9of72* patients and SMA patients based on published dataset from Sareen et al. 2018 ^1^; (B) RNA expression level of CDKN1A in iPSC-derived motor neurons from healthy control, C9of72 patients based on published datasets from Shi et al. 2018 ^2^.

**Supplementary Tables:**

**Table S1. Primers for semi-** **quantitative RT-PCR, qPCR, sequencing, cloning related to Gene editing**

| Name | Forward (Fwd) Primer Sequence | Reverse (Rev) Primer Sequence |
| --- | --- | --- |
| *5'JA AAVS1* | 5’ CTGCCGTCTCTCTCCTGAGTC 3’ | 5’ TTGTGCCCAGTCATAGCCGAAT 3’ |
| *3'JA AAVS1* | 5’ TTAGACATGCTCCCAGCCGATG 3’ | 5’ TACCCCGAAGAGTGAGTTTGCC 3’ |
| *GFP* | 5’ CTGGTCGAGCTGGACGGCGACG 3’ | 5’ CACGAACTCCAGCAGGACCATG 3’ |
| *TIA1g1* | 5’ GGATAAATGTTGGCGTGCTT 3’ | 5’ TACTCTGCATGCCTCAGGTG 3’ |
| *TIA1g2* | 5’ CCCAGGAGGTGGAGATTGTA 3’ | 5’ TAGCCAACCAGTTGACACCA 3’ |
| *TIA1g3* | 5’ TGTAATGTCTGGGCAACCAA 3’ | 5’ TGGGAAAACAATCTTTTGCTG 3’ |
| *Lin28g* | 5’ AGCGGGGACACTTTAGGATT 3’ | 5’ GGGTGCTGATAATTGGTGCT 3’ |
| *Human GAPDH* | 5’ ACCAGGAAATGAGCTTGACAAA 3’ | 5’ TCAAGAAGGTGGTGAAGCAGG 3’ |
| *Zebrafish nek6* | 5’ GATGGATGCTAAAGCCAGACAG GAC 3’ | 5’ TCAATCTTATGGCACAGCGAGA GC 3’ |
| *Zebrafish gapdh* | 5’ CCCATGTTTGTCATGGGTGT 3’ | 5’ GGTTGCTGTAACCGAACTCA 3’ |
| *Human PRT* | 5’ TGGGATTACACGTGTGAACCAAC 3’ | 5′GCTCTACCCTGTCCTCTACCGTCC3' |

**Table S2. List of antibodies, Related to Immunocytochemistry and Western blotting**

| Antibody | Isotype | Dilution | Source |
| --- | --- | --- | --- |
| OCT4 | Rabbit IgG | 1/400 | Santa Cruz |
| Cas9 | Rabbit IgG | 1/1000 | Abcam |
| TUJ1 | Chicken IgG | 1/1000 | Millipore |
| TBR1 | Rabbit IgG | 1/500 | Abcam |
| CTIP2 | Rat IgG | 1/300 | Abcam |
| Poly-PR | Rabbit IgG | 1/300 | Thermo Fisher Scientific (custom) |
| CRKL | Rabbit IgG | 1/1000 | Thermo Fisher Scientific |
| SNRK | Rabbit IgG | 1/1000 | Thermo Fisher Scientific |
| NEK6 | Rabbit IgG | 1/1000 | Atlas Antibodies |
| NEK6 (for zebrafish) | Rabbit IgG | 1/1000 | Abcam |
| NEK6 (for immunostaining) | Mouse IgG | 1/50 | Thermo Fisher Scientific |
| NeuN | Guinea pig IgG | 1/200 | Synaptic Systems |
| GFAP | Mouse IgG | 1/200 | Sigma-Aldrich |
| α-Tubulin | Mouse IgG | 1/5000 | Sigma-Aldrich |
| β-Actin | Mouse IgG | 1/5000 | Sigma-Aldrich |
| GAPDH | Mouse IgG | 1/5000 | Ambion |
| SV2 | Mouse IgG | 1/200 | DSHB |
| 53BP1 | Rabbit IgG | 1/750 | Cell Signaling |
| p53BP1 | Rabbit IgG | 1/750 | Cell Signaling |
| γH2AX | Rabbit IgG | 1/1000 | Cell Signaling |
| p21 | Rabbit IgG | 1/1000 | Cell Signaling |
| P53 | Rabbit IgG | 1/1000 | Cell Signaling |

**Table S3. Clinical information of PBSC sample donors**

| **Groups** | **PBMC code** | **Age** | **Gender** | **Side on onset** | **Diagnosis** |
| --- | --- | --- | --- | --- | --- |
| C9 patient | 1 | 65 | M | Spinal | Probable ALS |
| C9 patient | 2 | 63 | F | Bulbar | Possible ALS |
| C9 patient | 3 | 63 | F | Bulbar | Possible ALS |
| C9 patient | 4 | 59 | F | Bulbar | Suspected ALS |
| C9 patient | 5 | 55 | F | Bulbar | Possible ALS |
| C9 patient | 6 | 52 | M | Spinal | Probable ALS |
| C9 patient | 7 | 52 | M | Spinal | Probable ALS |
| C9 patient | 8 | 43 | F | Spinal | Probable ALS |
| C9 patient | 9 | 67 | M | Spinal | Possible ALS |
| C9 patient | 10 | 63 | M | Bulbar | Possible ALS |
| Non-C9 patient | S-1 | 57 | M | Spinal | Probable ALS |
| Non-C9 patient | S-2 | 57 | M | Bulbar | Possible ALS |
| Non-C9 patient | S-3 | 53 | M | Bulbar | Probable ALS |
| Non-C9 patient | S-4 | 60 | M | Bulbar | Probable ALS |
| Non-C9 patient | S-5 | 64 | M | Spinal | Possible ALS |
| Non-C9 patient | S-6 | 61 | F | Spinal | Suspected ALS |
| Non-C9 patient | S-7 | 58 | F | Spinal | Possible ALS |
| Non-C9 patient | S-8 | 54 | F | Spinal | Possible ALS |
| Non-C9 patient | S-9 | 49 | F | Spinal | Possible ALS |
| Non-C9 patient | S-10 | 52 | F | Spinal | Probable ALS |
| Control | C-1 | 58 | M |  |  |
| Control | C-2 | 60 | M |  |  |
| Control | C-3 | 53 | F |  |  |
| Control | C-4 | 56 | F |  |  |
| Control | C-5 | 59 | M |  |  |
| Control | C-6 | 55 | M |  |  |
| Control | C-7 | 68 | F |  |  |
| Control | C-8 | 50 | M |  |  |
| Control | C-9 | 67 | F |  |  |
| Control | C-10 | 56 | F |  |  |

Note: C9 patient: ALS patient that carrying C9orf72 mutations. Non-C9 cases do not have mutation in *C9orf 72, SOD1, TDP-43, FUS*; Diagnosis based on El Escorial World Federation of Neurology criteria.

**Table S4. Clinical and pathological information of post-mortem brain tissue donors**

| **Groups** | **Case n°** | **Age** | **Gender** | **Diagnosis** | **Disease duration** | **PMI** | **WB/IF** |
| --- | --- | --- | --- | --- | --- | --- | --- |
| C9 patient | C9-1 | 52 | m | ALS | 15 months | 6 | WB + IF |
| C9 patient | C9-2 | 58 | m | FTLD-TDP Type B | 36 months | 9 | WB |
| C9 patient | C9-3 | 58 | f | FTLD-TDP Type B, AGD | 48 months | 24 | WB |
| C9 patient | C9-4 | 55 | m | ALS, FTLD-TDP Type B | 30 months | 12 | WB |
| C9 patient | C9-5 | 57 | m | ALS | 19 months | 20 | WB |
| C9 patient | C9-6 | 48 | m | ALS | 18 months | 24 | WB |
| C9 patient | C9-7 | 49 | m | ALS, PART | 46 months | 24 | WB + IF |
| C9 patient | C9-8 | 57 | m | ALS, p-preAD | 17 months | n.d. | WB |
| Non-C9 patient | N-1 | 62 | f | FTLD-TDP Type C | 144 months | 12 | WB |
| Non-C9 patient | N-2 | 57 | f | ALS, FTLD-TDP Type B | 22 months | 24 | WB |
| Non-C9 patient | N-3 | 54 | m | ALS | 88 months | 6 | WB |
| Non-C9 patient | N-4 | 74 | m | ALS | 47 months | 24 | WB |
| Non-C9 patient | N-5 | 79 | f | FTLD-TDP Type A, ARTAG | 126 months | 24 | WB |
| Non-C9 patient | N-6 | 49 | m | ALS, PART | 45 months | 24 | WB |
| Non-C9 patient | N-7 | 58 | f | ALS, PART | 18 months | 24 | WB |
| Non-C9 patient | N-8 | 53 | m | ALS | 92 months | 24 | WB |
| Control | Con-1 | 59 | m | Control, I | n.a. | 72 | WB |
| Control | Con-2 | 67 | f | Control, ARTAG | n.a. | n.d. | WB |
| Control | Con-3 | 64 | f | Control, AGD, I | n.a. | n.d. | WB |
| Control | Con-4 | 68 | m | Control | n.a. | 48 | WB |
| Control | Con-5 | 68 | f | Control, I, MI, SVD | n.a. | 24 | WB |
| Control | Con-6 | 66 | m | Control | n.a. | 6 | WB + IF |
| Control | Con-7 | 64 | f | Control, MI, H | n.a. | 48 | WB |
| Control | Con-8 | 54 | m | Control, GB | n.a. | 24 | IF |

Note: C9 patient: ALS or FTLD patient that have C9orf72 mutations; Non-C9 patient: ALS or FTLD patient that do not have C9orf72 mutations; Control: no neurological disorder case. The diagnosis was based on clinical assessment according to the consensus criteria ^3–5^. This table gives information on age (= age at death), gender, diagnosis, the presence of the *C9orf72* mutation, disease duration (months), PMI (hours) and application of the tissue (WB/IF). Abbreviations: f = female; m = male; ALS = amyotrophic lateral sclerosis; FTLD = frontotemporal lobar degeneration; control = non neurodegenerative disease control; AGD = argyrophilic grain disease; ARTAG = aging-related tau astrogliopathy; GB = Guillian-Barre syndrome; H = hemorrhage; I = infarction; MI = microinfarction; n.a. = not applicable; n.d. = not determined; p-preAD = pathologically defined preclinical AD; PART = primary age-related tauopathy; SVD = small vessel disease; PMI = post-mortem interval.

**References**

1. Sareen D, O’Rourke JG, Meera P, et al. Targeting RNA foci in iPSC-derived motor neurons from ALS patients with a C9ORF72 repeat expansion. *Sci Transl Med*. 2013;5(208):208ra149. doi:10.1126/scitranslmed.3007529

2. Shi Y, Lin S, Staats KA, et al. Haploinsufficiency leads to neurodegeneration in C9ORF72 ALS/FTD human induced motor neurons. *Nat Med*. 2018;24(3):313-325. doi:10.1038/nm.4490

3. Brooks BR, Miller RG, Swash M, Munsat TL. El Escorial revisited: Revised criteria for the diagnosis of amyotrophic lateral sclerosis. *Amyotroph Lateral Scler*. 2000;1(5):293-299. doi:10.1080/146608200300079536

4. de Carvalho M, Dengler R, Eisen A, et al. Electrodiagnostic criteria for diagnosis of ALS. *Clin Neurophysiol*. 2008;119(3):497-503. doi:10.1016/j.clinph.2007.09.143

5. De Carvalho M, Swash M. Awaji diagnostic algorithm increases sensitivity of El Escorial criteria for ALS diagnosis. *Amyotroph Lateral Scler*. 2009;10(1):53-57. doi:10.1080/17482960802521126

6. Ordov??s L, Boon R, Pistoni M, et al. Efficient recombinase-mediated cassette exchange in hPSCs to study the hepatocyte lineage reveals AAVS1 locus-mediated transgene inhibition. *Stem Cell Reports*. 2015;5(5):918-931. doi:10.1016/j.stemcr.2015.09.004

7. Takahashi K, Tanabe K, Ohnuki M, et al. Induction of Pluripotent Stem Cells from Adult Human Fibroblasts by Defined Factors. *Cell*. 2007;107(5):861-872. doi:10.1016/j.cell.2007.11.019

8. Selvaraj BT, Livesey MR, Zhao C, et al. C9ORF72 repeat expansion causes vulnerability of motor neurons to Ca2+-permeable AMPA receptor-mediated excitotoxicity. *Nat Commun*. 2018;9(1):347. doi:10.1038/s41467-017-02729-0

9. Shi Y, Kirwan P, Livesey FJ. Directed differentiation of human pluripotent stem cells to cerebral cortex neurons and neural networks. *Nat Protoc*. 2012;7(10):1836-1846. doi:10.1038/nprot.2012.116

10. García-León JA, Cabrera-Socorro A, Eggermont K, et al. Generation of a human induced pluripotent stem cell-based model for tauopathies combining three microtubule-associated protein TAU mutations which displays several phenotypes linked to neurodegeneration. *Alzheimer’s Dement*. 2018;14(10):1261-1280. doi:10.1016/j.jalz.2018.05.007

11. Claes C, Van Den Daele J, Boon R, et al. Human stem cell-derived monocytes and microglia-like cells reveal impaired amyloid plaque clearance upon heterozygous or homozygous loss of TREM2. *Alzheimer’s Dement*. 2019;15(3):453-464. doi:10.1016/j.jalz.2018.09.006

12. Bustin SA, Benes V, Garson JA, et al. The MIQE Guidelines: Minimum Information for Publication of Quantitative Real-Time PCR Experiments. *Clin Chem*. 2009;55(4):611-622. doi:10.1373/clinchem.2008.112797

13. Vandoorne T, Veys K, Guo W, et al. Differentiation but not ALS mutations in FUS rewires motor neuron metabolism. *Nat Commun*. 2019;10(1):4147. doi:10.1038/s41467-019-12099-4

14. Guo W, Naujock M, Fumagalli L, et al. HDAC6 inhibition reverses axonal transport defects in motor neurons derived from FUS-ALS patients. *Nat Commun*. 2017;8(1):861. doi:10.1038/s41467-017-00911-y

15. Tharkeshwar AK, Trekker J, Vermeire W, et al. A novel approach to analyze lysosomal dysfunctions through subcellular proteomics and lipidomics: the case of NPC1 deficiency. *Sci Rep*. 2017;7(1):41408. doi:10.1038/srep41408

16. Van Leene J, Han C, Gadeyne A, et al. Capturing the phosphorylation and protein interaction landscape of the plant TOR kinase. *Nat Plants*. 2019;5(3):316-327. doi:10.1038/s41477-019-0378-z

17. Maia TM, Staes A, Plasman K, et al. Simple Peptide Quantification Approach for MS-Based Proteomics Quality Control. *ACS Omega*. 2020;5(12):6754-6762. doi:10.1021/acsomega.0c00080

**Supplementary Methods:**

**Induced pluripotent stem cells and culture**

The normal donor iPSCs used were either generated in house (BJ1-iPSC^6,7^) or purchased from Sigma IPSC00028 iPSC). ALS patient-derived hiPSCs and their isogenic controls were kind gifts from Edinburgh University^8^. All hiPSCs were maintained on Corning Matrigel Matrix (734-0269, Corning) in Essential 8^TM^ medium (A1517001, GibcoTM) with 1000U/ml penicillin–streptomycin. Colonies were routinely passaged with 0.5 mM EDTA (15575-020, Invitrogen) in Dulbecco’s phosphate-buffered saline (DPBS). Cultures were routinely analyzed by PCR for mycoplasma contamination. All studies using human iPSCs were approved by the Human Ethics Committee at the University Hospital, Gasthuisberg, KU Leuven, Belgium.

**Differentiation of cortical neurons from hiPSCs**

Cortical neurons were generated based on the Shi et al protocol ^9^, and as previously published by us ^10^. The hiPSC clones were dissociated and plated as single cells on Matrigel-coated (BD) plates in mTESR1 medium (StemCell Technologies) containing 10 μM of Y-27632 Rho-Associated Kinase (ROCK) inhibitor (ROCKi; Calbiochem). Once the cell culture reached 90-95% confluence, change the culture medium to neuronal induction medium that made by a 1:1 mixture of of N2- and B27-containing media (N2 medium was made by mixing DMEM/F12, N2, 5 mg/mL insulin, 1x nonessential amino acids, 1x 2-mercaptoethanol, 1x sodium pyruvate; B27 was made by mix Neurobasal, B27 without retinoic acid, 1x glutamax, and 1x penicillin/streptomycin. Insulin was purchased from Sigma and all other reagents were purchased from Life Technologies) supplemented with SB431542 10 mM (Tocris) and LDN193189 1 mM (Miltenyi Biotec) with daily medium change for 10 days. Once a uniform neuroepithelial cell layer was formed, neuroepithelial cell were dissociated by Dispase and replated on Matrigel (BD) and cultured in N2B27 medium supplemented with 20 ng/ml FGF-2 (R&D Systems) to purify the neuroepithelial cells. Afterwards, neuroepithelial cells were purified two more times (each after 4–5 days) to obtain > 90% pure neural progenitor cells (NPCs) cells. Around DIV 30, NPCs were split to single cells by Accutase and cryopreserved. For final maturation, NPCs were cultured on Matrigel-coated plates and replated at DIV 45-60 on poly-ornithine and laminin-coated plastic dishes and cultured in N2B27 medium and maintained until up to DIV100 with medium change twice a week.

**Recombinase-mediated cassette exchange of the inducible Cas9 sequence in hiPSCs**

As described before^6^ , an FRT-flanked donor cassette had previously been inserted into the *AAVS1* locus of BJ1 and Sigma IPSC00028 cells. Here we used recombinase-mediated cassette exchange to establish the iCas9 hiPSCs. The FRT-flanked donor plasmid is described in Figure 1A. The Cas9 cDNAs were purchased from OriGene Technologies (Rockville, USA). G418 (100 ug/ml) and 0.5 μM1 ‐(2‐deoxy‐2‐ﬂuoro‐beta‐D‐arabinofuranosyl) ‐5‐iodouracil (FIAU) were used to select for correctly recombined colonies. Correct integration of the cassette was demonstrated by 3’ and 5’ junction assay PCR. In addition, we used digital droplet PCR to demonstrate that only 1 cassette was present in the cells. Other QC studies were performed as described previously^6,11^.

**Digital droplet PCR**

Genomic DNA was isolated using a DNeasy kit (Qiagen) and sequencing was performed by LGC Genomics (Berlin, Germany). HAEIII restriction enzyme (New England Biolabs) was used for the random digestion of the genomic DNAs for 2 hours. Cas9 probe were designed in house and synthesized in IDT (Germany). AP3B1 probe (dHsaCP1000001 Bio-rad) and eGFP probe (Mr00660654_cn ThermoFisher Scientific) are commercially available and were used as the reference gene. Bio-Rad ddPCR mix was used for PCR amplification. PCR mix were loaded into individual wells of disposable droplet generator cartridge (Bio-Rad). QX200 droplet generator (Bio-Rad) was used for generating droplets. Once droplets were generated, a thermal cycler was used for the PCR reaction (40 cycles). Finally, the PCR plates were read by a QX200 droplet reader (Bio-Rad).

**RT/Real-time PCR**

Total RNA of hiPSC-derived cortical neurons and zebrafish embryos (at 30 hpf) were isolated by using the RNeasy kit (Qiagen) and reverse transcription was performed using SuperScript® III First-Strand Synthesis SuperMix (Invitrogen) for both qRT-PCR and RT-PCR. qRT-PCR was performed using SYBR® Green PCR Master Mix (Applied Biosystems™) on the 7500 Step OnePlus™ Real-Time PCR System (Applied BiosystemsTM). All samples were run in triplicate and relative quantiﬁcation was done using the ΔΔCt method with normalization to reference genes. RT-PCR was performed by using a 96-well thermal cycler (Thermo Fisher Scientific) with optimized program. RT-PCR was done using DreamTaq Green Master Mix (Thermo Fisher Scientific), according to the manufacturer’s instructions. RT-PCR reactions were analyzed on a 2% agarose gel and visualized by MIDORI Green staining (Nippon Genetics).

For hiPSC-derived cortical neurons, the NEK6 RT-primer set was purchased from Santa Cruz (sc-61172-PR). Cas9 real-time primers was purchased from System Biosciences (CAS9-PR-1-SBI). For zebrafish, the nek6 and gapdh RT-primers were designed in house and synthesized by IDT (Integrated DNA Technologies). A list of primers can be found in Supplementary Table 1.

**TaqMan Real-Time PCR on human peripheral blood mononuclear cells (PBMC)**

Peripheral blood mononuclear cells (PBMCs) were obtained from 10 healthy individuals, 10 sporadic ALS patients without known mutations and 10 ALS patients carrying a *C9orf72* mutation, approved by the Human Ethics Committee at the University Hospital, Gasthuisberg, KU Leuven, Belgium. The mean age of patients in the three groups was similar, and the number of males and females was also similar. The clinical data from these individuals are described in Supplementary Table 3.

Total RNA was extracted from PBMCs using TRI Reagent® (Sigma Aldrich/Merck) according to the manufacturer’s instructions. Reverse transcription was performed as above with 1 ug RNA. All samples were processed at the same time, the resulting cDNA diluted 1:20 in nuclease-free water, and 5 µl was used for real-time PCR. Real-time PCR reactions were performed on a 384-well format using the ViiA7 (Applied Biosystems/Thermo Fisher). All assays were probe-based allowing multiplexing, the genes of interest [*NEK1* ([Hs01583138_m1](https://www.thermofisher.com/taqman-gene-expression/product/Hs01583138_m1?CID=&ICID=&subtype=)), *NEK6* (Hs01032395_m1), *NEK7* (Hs00370356_m1)] and stable reference genes [*HPRT* (Hs02800695_m1) and *RPLPO* (Hs00420895_gH)] (all Applied Biosystems/Thermo Fisher). The assays were validated, and PCR efficiency was 100% +/- 10%. Real-time reactions were run in triplicate using TaqMan™ Fast Universal PCR Master Mix (2X), no AmpErase™ UNG (Applied Biosystems/Thermo Fisher). Collected data were analyzed using qbase^plus^ (Biogazelle), according to standards of the MIQE guidelines^12^.

**RNA sequencing**

RNA sequencing was performed by the Nucleomics Core Facility (VIB, Leuven, Belgium) as described before^13^.

**Unassisted delivery of ASOs**

Locked Nucleic Acid (LNA™) oligonucleotides for NEK6 and SNRK were purchased from Exiqon (Vedbaek, Denmark). DIV72 iPSC-derived cortical neurons were incubated with the LNAs, dissolved in sterilized water, at a ﬁnal concentration of 50nM for 1 week prior to axonal transport analysis or other downstream analyses.

**Cell viability test**

Resazurin (R7017, Sigma) was added to the cells in neuron maintenance medium (NMM) at a concentration of 1µg/ml. Cells were incubated for 1 h in 37°C with 5%CO2. Absorbance was evaluated using 540 nm excitation and 590 nm emission settings.

**Immunofluorescence staining**

For immunofluorescence analysis, cells plated on coverslips were ﬁxed in 4% paraformaldehyde for 20 min at room temperature, and then washed with PBS. Permeabilization was done for 30 min and blocking was done for 1 h using PBS containing 0.2% Triton X-100 (Acros Organics) and 5% donkey serum (Sigma). Cells were incubated overnight at 4°C in blocking buffer (2% donkey serum) containing the different primary antibodies (Abs). After washing with PBS, cells were incubated with secondary antibodies (Invitrogen) for 1 h at room temperature. Fluorescent and bright ﬁeld micrographs were captured using a Zeiss Axio Imager M1 microscope (Carl Zeiss) equipped with an AxioCam MRc5 (bright ﬁeld, Carl Zeiss) or a monochrome AxioCam Mrm camera (ﬂuorescence, Carl Zeiss). Primary antibodies are listed in Supplementary Table 2.

**Western blotting**

For Western blot analysis, cells and zebrafish embryos (6 hpf) were collected on dry ice and maintained at −80°C until further processing. Samples were hydrolyzed in RIPA buffer (containing 50 mM Tris, 150 mM NaCl, 1% (vol/ vol) NP40, 0.5% sodium deoxycholate (wt/vol), 0.1% SDS (wt/vol) complemented with protease inhibitors (Complete, Roche Diagnostics, pH 7.6). Protein concentrations were determined using the microBCA kit (Thermo Fisher Scientiﬁc Inc.) according to the manufacturer’s instructions. Western blotting was performed as described before^14^. Optical densities were determined using the integrated density measurement tool of ImageJ (NIH). Primary antibodies are listed in Supplementary Table 2.

**Proteomics**

Peptides from cortical neuronal pellets were prepared as described before^15^. A part (100 µl) was dried completely and used for shotgun analysis, while the rest was used for phosphopeptide enrichment as described^15–17^. Data analysis of the shotgun and phosphoproteomics data was performed with MaxQuant (version 1.6.11.0) using the Andromeda search engine with default search settings. Spectra were searched against the human proteins in the Swiss-Prot Reference Proteome database (version January 2020). The mass tolerance for precursor and fragment ions was set to 4.5 and 20 ppm, while the enzyme specificity was set as C-terminal to arginine and lysine. Variable modifications were set to oxidation of methionine residues, acetylation of protein N-termini and phosphorylation of serine, threonine or tyrosine residues, while carbamidomethylation of cysteine residues was set as fixed modification. Matching between runs was enabled with a matching time window of 0.7 minutes and an alignment time window of 20 minutes. Only proteins with at least one unique or razor peptide were retained MaxLFQ algorithm integrated in the MaxQuant software was used to quantify the proteins that had a minimum ratio count of two unique or razor peptides. A two-way ANOVA test was performed to compare the intensities of the proteins in the Condition group (Ctrl vs. KD) to reveal proteins in which the expression level was significantly regulated. For each protein, this test calculated a p-value (-log p-value) for Condition. For the analysis of the phosphoproteomics data, the phospho (STY)sites file was loaded in the Perseus software (version 1.6.2.1). Reverse hits were removed, the site table was expanded, the intensity values were log2 transformed and the median was subtracted. Replicate samples were grouped, phosphosites with less than three valid values in at least one group were removed and missing values were imputed from a normal distribution around the detection limit leading to a list of quantified phosphopeptides that was used for further data analysis. Then, t-tests were performed (FDR=0.05 and s0=1) to compare phosphopeptide intensities in the different sample types. The mass spectrometry proteomics data will be deposited to the ProteomeXchange Consortium via the PRIDE partner.

**Science illustrations**

The graphs that presented in this manuscript have adapted some images from Smart service medical art (https://smart.servier.com/), BioRender (https://biorender.com/) and somersault 1824 (https://www.somersault1824.com/)

**References**

1. Sareen D, O’Rourke JG, Meera P, et al. Targeting RNA foci in iPSC-derived motor neurons from ALS patients with a C9ORF72 repeat expansion. *Sci Transl Med*. 2013;5(208):208ra149. doi:10.1126/scitranslmed.3007529

2. Shi Y, Lin S, Staats KA, et al. Haploinsufficiency leads to neurodegeneration in C9ORF72 ALS/FTD human induced motor neurons. *Nat Med*. 2018;24(3):313-325. doi:10.1038/nm.4490

3. Brooks BR, Miller RG, Swash M, Munsat TL. El Escorial revisited: Revised criteria for the diagnosis of amyotrophic lateral sclerosis. *Amyotroph Lateral Scler*. 2000;1(5):293-299. doi:10.1080/146608200300079536

4. de Carvalho M, Dengler R, Eisen A, et al. Electrodiagnostic criteria for diagnosis of ALS. *Clin Neurophysiol*. 2008;119(3):497-503. doi:10.1016/j.clinph.2007.09.143

5. De Carvalho M, Swash M. Awaji diagnostic algorithm increases sensitivity of El Escorial criteria for ALS diagnosis. *Amyotroph Lateral Scler*. 2009;10(1):53-57. doi:10.1080/17482960802521126

6. Ordov??s L, Boon R, Pistoni M, et al. Efficient recombinase-mediated cassette exchange in hPSCs to study the hepatocyte lineage reveals AAVS1 locus-mediated transgene inhibition. *Stem Cell Reports*. 2015;5(5):918-931. doi:10.1016/j.stemcr.2015.09.004

7. Takahashi K, Tanabe K, Ohnuki M, et al. Induction of Pluripotent Stem Cells from Adult Human Fibroblasts by Defined Factors. *Cell*. 2007;107(5):861-872. doi:10.1016/j.cell.2007.11.019

8. Selvaraj BT, Livesey MR, Zhao C, et al. C9ORF72 repeat expansion causes vulnerability of motor neurons to Ca2+-permeable AMPA receptor-mediated excitotoxicity. *Nat Commun*. 2018;9(1):347. doi:10.1038/s41467-017-02729-0

9. Shi Y, Kirwan P, Livesey FJ. Directed differentiation of human pluripotent stem cells to cerebral cortex neurons and neural networks. *Nat Protoc*. 2012;7(10):1836-1846. doi:10.1038/nprot.2012.116

10. García-León JA, Cabrera-Socorro A, Eggermont K, et al. Generation of a human induced pluripotent stem cell-based model for tauopathies combining three microtubule-associated protein TAU mutations which displays several phenotypes linked to neurodegeneration. *Alzheimer’s Dement*. 2018;14(10):1261-1280. doi:10.1016/j.jalz.2018.05.007

11. Claes C, Van Den Daele J, Boon R, et al. Human stem cell-derived monocytes and microglia-like cells reveal impaired amyloid plaque clearance upon heterozygous or homozygous loss of TREM2. *Alzheimer’s Dement*. 2019;15(3):453-464. doi:10.1016/j.jalz.2018.09.006

12. Bustin SA, Benes V, Garson JA, et al. The MIQE Guidelines: Minimum Information for Publication of Quantitative Real-Time PCR Experiments. *Clin Chem*. 2009;55(4):611-622. doi:10.1373/clinchem.2008.112797

13. Vandoorne T, Veys K, Guo W, et al. Differentiation but not ALS mutations in FUS rewires motor neuron metabolism. *Nat Commun*. 2019;10(1):4147. doi:10.1038/s41467-019-12099-4

14. Guo W, Naujock M, Fumagalli L, et al. HDAC6 inhibition reverses axonal transport defects in motor neurons derived from FUS-ALS patients. *Nat Commun*. 2017;8(1):861. doi:10.1038/s41467-017-00911-y

15. Tharkeshwar AK, Trekker J, Vermeire W, et al. A novel approach to analyze lysosomal dysfunctions through subcellular proteomics and lipidomics: the case of NPC1 deficiency. *Sci Rep*. 2017;7(1):41408. doi:10.1038/srep41408

16. Van Leene J, Han C, Gadeyne A, et al. Capturing the phosphorylation and protein interaction landscape of the plant TOR kinase. *Nat Plants*. 2019;5(3):316-327. doi:10.1038/s41477-019-0378-z

17. Maia TM, Staes A, Plasman K, et al. Simple Peptide Quantification Approach for MS-Based Proteomics Quality Control. *ACS Omega*. 2020;5(12):6754-6762. doi:10.1021/acsomega.0c00080
